# Seeking Solutions for Inclusively Economic, Rapid, and Safe Molecular Detection of Respiratory Infectious Diseases: Comprehensive Review from Polymerase Chain Reaction Techniques to Amplification-Free Biosensing

**DOI:** 10.3390/mi16040472

**Published:** 2025-04-15

**Authors:** Yaping Xie, Zisheng Zong, Qin Jiang, Xingxing Ke, Zhigang Wu

**Affiliations:** 1 State Key Laboratory of Intelligent Manufacturing Equipment and Technology, School of Mechanical Science and Engineering, Huazhong University of Science and Technology, Wuhan 430074, China; yapingx@sansure.com.cn (Y.X.); zongzs@hust.edu.cn (Z.Z.); jiangqin@hust.edu.cn (Q.J.); xxke@fzu.edu.cn (X.K.); 2 Sansure Biotech Inc., Changsha 410205, China; 3 School of Mechanical Engineering and Automation, Fuzhou University, Fuzhou 350108, China

**Keywords:** molecular detection of respiratory infectious diseases, polymerase chain reaction, isothermal amplification, amplification-free biosensing, full scene solution, tiered diagnosis

## Abstract

Frequent outbreaks of respiratory infectious diseases, driven by diverse pathogens, have long posed significant threats to public health, economic productivity, and societal stability. Respiratory infectious diseases are highly contagious, characterized by short incubation periods, diverse symptoms, multiple transmission routes, susceptibility to mutations, and distinct seasonality, contributing to their propensity for outbreaks. The absence of effective antiviral treatments and the heightened vulnerability of individuals with weakened immune systems make them more susceptible to infection, with severe cases potentially leading to complications or death. This situation becomes particularly concerning during peak seasons, such as influenza outbreaks. Therefore, early detection, diagnosis, and treatment are critical, alongside the prevention of cross-infection, ensuring patient safety, and controlling healthcare costs. To address these challenges, this review aims to identify a comprehensive, rapid, safe, and cost-effective diagnostic approach for respiratory infectious diseases. This approach is framed within the existing hierarchical healthcare system, focusing on establishing diagnostic capabilities at hospitals, community, and home levels to effectively tackle the above issues. In addition to PCR and isothermal amplification, the review also explores emerging molecular diagnostic strategies that may better address the evolving needs of respiratory disease diagnostics. A key focus is the transition from amplification technologies to amplification-free biosensing approaches, with particular attention given to their potential for home-based testing. This shift seeks to overcome the limitations of conventional amplification methods, particularly in decentralized and home diagnostics, offering a promising solution to enhance diagnostic speed and safety during outbreaks. In the future, with the integration of AI technologies into molecular amplification technologies, biosensors, and various application levels, the inclusively economic, rapid, and safe respiratory disease diagnosis solutions will be further optimized, and their accessibility will become more widespread.

## 1. Introduction

The widespread prevalence and outbreaks of respiratory infectious diseases, such as the SARS outbreak in 2003 [1], the MERS outbreak in 2012 [2], and the COVID-19 pandemic in 2019 [3], have had a profound impact on human health, economic development, and social stability. In particular, the COVID-19 pandemic, which began in early 2020, affected over 200 countries worldwide. As of January 2025, more than 777 million confirmed cases and 7.08 million deaths have been reported globally [4]. These outbreaks have not only caused severe economic disruptions [5], but also widespread suffering, characterized by a complex mix of emotions—denial, panic, insecurity, uncertainty, mistrust, loneliness, heroism (and cowardice), feelings of punishment, scapegoating, hedonistic excess, discouragement, and even mental breakdowns [6].

Since the outbreak of COVID-19, the landscape of respiratory infectious diseases has shifted dramatically, with these diseases becoming increasingly prevalent and posing significant public health challenges. Respiratory infections [7] are often caused by a wide array of pathogens, exhibit complex transmission dynamics, and affect a broad spectrum of the population, particularly those with underlying health conditions. The rapid and widespread transmission, coupled with high infectiousness, makes these diseases difficult to control in time. Moreover, the lack of effective and specific treatments for many respiratory infections exacerbates their impact, contributing to elevated morbidity and mortality rates.

The above challenges are particularly pronounced during peak seasons for respiratory diseases such as influenza when the demand for healthcare services surges. The limitations of at-home testing, coupled with insufficient community healthcare resources, often result in bottlenecks in the healthcare system, as large numbers of patients seek care at hospitals [8]. Such an overwhelming influx results in shortages of diagnostic resources, significantly heightening the risk of nosocomial infections within healthcare facilities. Moreover, the increasing need for critical medical resources such as ICU beds, ventilators, and medications intensifies the strain on healthcare systems, resulting in delays in treatment, which in turn worsens patient outcomes and further escalates disease transmission, ultimately raising mortality rates [9].

To effectively prevent and control respiratory infectious diseases, establishing a comprehensive “rapid, safe, and cost-effective” solution is essential. First, a rapid response mechanism is key to containing the spread of an outbreak. Early detection, achieved through proactive monitoring and preventive measures, is essential for identifying potential infections before they spread. Early diagnosis follows, requiring the rapid identification and confirmation of cases at the initial stages of illness. The final component, early treatment, focuses on intervening within the patient’s golden therapeutic window to improve outcomes and prevent disease escalation. Such rapid responses are fundamental to reducing the burden of infectious diseases and preventing widespread outbreaks [10]. Second, ensuring a safe clinical environment is fundamental to preventing the further spread of an outbreak. Implementing stratified diagnosis and treatment based on disease severity enables appropriate care while minimizing the risk of cross-infection. This approach reduces transmission among patients, between patients and their families, and between patients and healthcare workers, effectively lowering the incidence of hospital-acquired infections. By protecting both patients and medical staff, it mitigates overall transmission risks within the healthcare system, enhancing safety and reducing the likelihood of further outbreak escalation [11]. Third, economic factors play a crucial role in the sustainability of epidemic prevention and control efforts. In addressing the disease, reducing the time, transportation, and treatment costs caused by diagnostic and treatment delays not only alleviates the financial burden on patients but also reduces the overall economic pressure on society. Additionally, by optimizing resource allocation and streamlining healthcare processes, timely and efficient disease prevention and treatment can be ensured, minimizing economic disruption. This economically driven approach enhances the sustainability and cost-effectiveness of epidemic control measures [12]. Recent public health events, including the COVID-19 pandemic, have highlighted significant flaws in the existing systems for early detection, diagnosis, treatment, and healthcare safety, particularly in preventing cross-infection. These deficiencies have resulted in suboptimal epidemic control and have had a profound economic impact.

**Figure 1:**
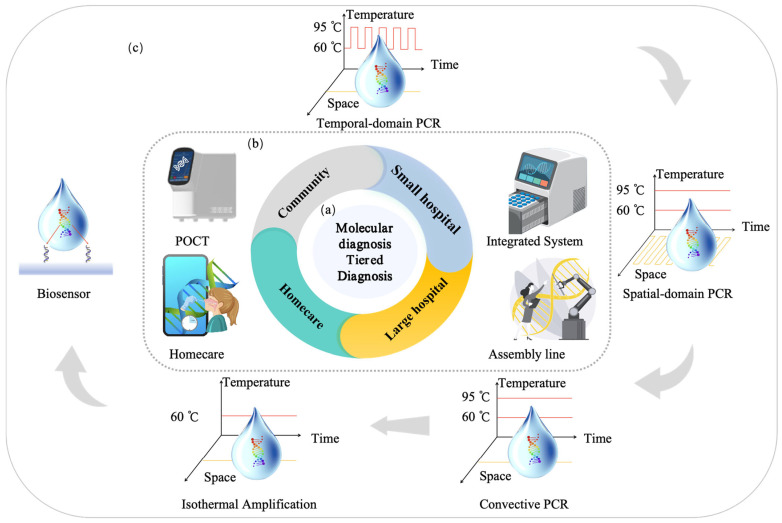
Comprehensive molecular diagnosis and treatment solution for respiratory infectious diseases. (**a**) Hierarchical diagnosis and treatment scenarios for infectious diseases; (**b**) solutions for different scenarios; and (**c**) overview of amplification and amplification-free methods.

The existing tiered healthcare model [13] can be adapted to meet the core demands of respiratory infectious disease prevention and control—speed, safety, and cost-effectiveness. Within this framework, the roles of different healthcare levels are further refined based on the characteristics of respiratory infections, while their capabilities are strengthened to enhance overall efficiency, as shown in Figure 1a. At the household level [14], establishing home testing capabilities and strengthening disease prevention and control measures help with early detection, enhancing the safety and timeliness of testing. At the community level, enhancing the use of POCT (point-of-care testing) technology [15] facilitates early diagnosis, improving the speed and safety of diagnosis and treatment while effectively reducing healthcare costs. At the hospital level, increasing batch testing capacity ensures timely treatment [16], while also lowering testing costs. Additionally, smaller hospitals should enhance their ability to perform small-batch, multi-sample testing and take on referral tasks within the tiered healthcare system, ensuring that patients receive timely and effective care, further reducing healthcare costs [17]. This tiered healthcare model, through effective stratification and management, ensures that patients are appropriately treated across households, communities, smaller hospitals, and large hospitals, thereby reducing the risk of cross-infection. Early treatment significantly reduces the medical costs associated with delayed diagnoses while also cutting patients’ time and transportation costs, ultimately optimizing the allocation of medical resources. Furthermore, this model accelerates the response and prevention efforts for respiratory infectious diseases, helping to alleviate the broader socio-economic impact of epidemics. That is, tiered healthcare strategies offer an effective means to achieve rapid, safe, and cost-effective solutions for addressing respiratory infectious diseases.

To achieve a tiered diagnosis and treatment system for respiratory infectious diseases and meet the testing needs across hospitals, communities, and home care settings, this review proposes a stratified diagnostic framework based on PCR technology, integrated with biosensors, as illustrated in Figure 1b. First, leveraging conventional PCR laboratory solutions [18], the design of high-throughput, fully automated rapid molecular diagnostic pipelines, or intelligent molecular diagnostic laboratories could reduce turnaround time (TAT) [19] and molecular testing costs. This ensures that hospitals can scale up to rapidly and automatically perform large-scale molecular testing for infectious diseases. Additionally, medium-throughput molecular diagnostic platforms will be designed to accommodate the diverse diagnostic requirements of various respiratory infectious disease samples, providing small hospitals with the capacity to process complex samples in smaller batches. At the community level, existing PCR technologies will be optimized to meet the design requirements of POCT devices [20]. By integrating nucleic acid extraction, amplification, and detection into a single cartridge using microfluidic technology, this approach ensures high sensitivity and specificity, overcoming the limitations of traditional PCR molecular laboratories. This streamlined process, from sample collection to result reporting, makes the system suitable for use by community healthcare providers [21]. Furthermore, the development of convective PCR technology, isothermal amplification, and amplification-free biosensors will simplify molecular diagnostic technologies for home use. The simplified biosensor technology features easy operation, reduced reagent complexity, and no amplification requirement, which will enhance the user experience, prevent aerosol contamination, and effectively reduce the occurrence of false positives. The diagnostic reagents will be lyophilized and stored at room temperature in test cartridges, making them accessible and easy to operate with high precision in ordinary household environments. In summary, as the diagnostic setting shifts from large hospitals to home applications, the demand for faster testing, higher efficiency, and ease of operation increases, while the need for high throughput decreases.

The reason why we chose to design the tiered diagnostic and treatment solution based primarily on PCR technology is that PCR, with its exceptional sensitivity, specificity, and convenience, remains the “gold standard” for pathogen detection, early diagnosis of infectious diseases, and epidemiological surveillance [22]. Additionally, incorporating amplification techniques and biosensing technologies as key tools for future development is driven by the fact that isothermal amplification technologies [23] and molecular biosensors [24], due to their rapid detection process, simple operation, and lack of need for complex equipment, are increasingly becoming important supplementary tools in infectious disease diagnostics, particularly in resource-limited settings.

In the aforementioned tiered diagnosis and treatment framework for respiratory diseases, different scenarios and healthcare levels have varying demands for molecular diagnostic technologies in terms of efficiency, accuracy, cost, ease of operation, and environmental adaptability. Therefore, it is necessary to conduct a detailed analysis of PCR technology, isothermal amplification techniques, and biosensor technologies across these key dimensions to ensure the selection of the most suitable, feasible, and practically applicable technical solutions for each scenario.

As shown in Figure 1c, we explore the development of technologies such as PCR, isothermal amplification, and biosensors, based on the analysis of time and spatial dimensions. The evolution of molecular diagnostic technologies follows this trajectory: initially relying on time-dependent PCR, which then evolved into spatial-domain PCR, followed by convective PCR that integrates both time and spatial factors; next, the technology transitioned to a simplified isothermal amplification method, which also unifies time and space; finally, the development advanced to biosensors that completely eliminate the amplification process. This evolution reflects the continuous effort to optimize molecular diagnostic technologies, shifting from complex, time-dependent methods to simpler, amplification-free systems. The paper will review the development and characteristics of the aforementioned technologies, as well as their applicability in different scenarios, aiming to construct an economical, safe, and rapid detection solution for respiratory infections. With the advancement of artificial intelligence (AI) technologies, future AI applications will empower PCR detection systems, enabling dynamic control of PCR fundamental technologies, systems, and reaction programs, as well as intelligent judgment and quality management. Furthermore, AI will optimize detection performance through the construction of large models based on detection data. At the same time, AI will enhance the performance of biosensors by deeply analyzing sensor detection data, improving detection sensitivity and accuracy. Finally, AI will play a significant role in managing application scenarios. These innovations will drive the optimization and practical application of safe, economical, and efficient solutions for the detection of respiratory infectious diseases.

## 2. Analysis of Molecular Diagnostic Technologies

Currently, commonly used molecular diagnostic methods for respiratory infectious diseases include PCR, isothermal amplification, and biosensors. For PCR-based detection, enhancing the efficiency of thermal cycling can significantly accelerate diagnostic speed. Key factors influencing thermal cycling efficiency include heat transfer and thermal resistance. This study employs a thermal resistance model, in conjunction with the different PCR technologies and isothermal amplification methods illustrated in Figure 1c, to analyze heat transfer efficiency. Furthermore, a summary of the efficiency of typical PCR-based molecular amplification technologies is provided. Additionally, the review offers an analysis of biosensors, ranging from amplification-based to amplification-free methods, in the context of molecular diagnostics.

### 2.1. Temporal-Domain PCR

**Figure 2:**
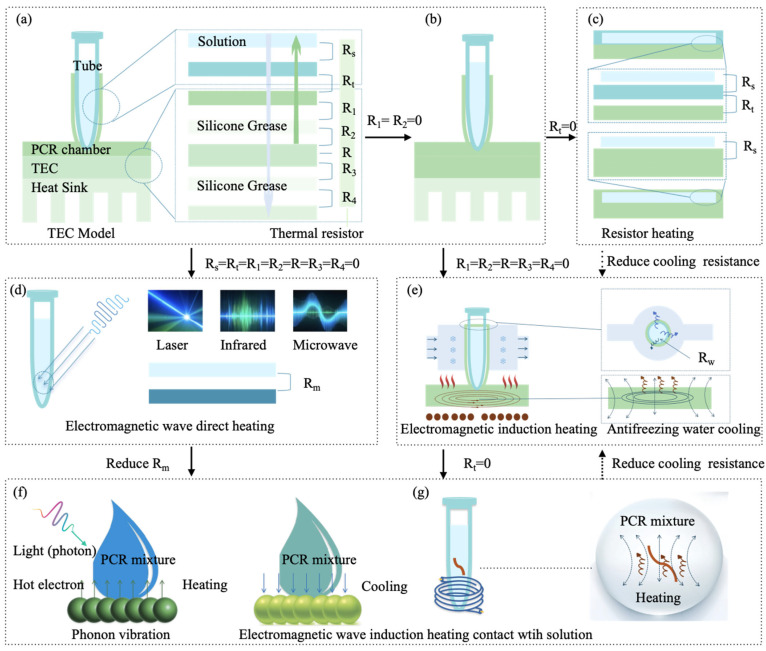
Temporal-domain PCR. (**a**) Traditional thermoelectric cooler; (**b**) integration of TEC and PCR chamber; (**c**) resistive heating; (**d**) direct heating via electromagnetic waves; (**e**) indirect heating via electromagnetic waves with heat removal through flow; (**f**) direct heating and cooling via electromagnetic wave-induced nanoparticles [25] (**g**) electromagnetic wave-induced metal heating.

Temporal-domain PCR refers to the process where during the heating and cooling phases of PCR, the spatial position of the reaction mixture remains constant, while the temperature cycles periodically in response to time (Figure 1c). The following section analyzes several commonly used temporal-domain PCR methods (Figure 2).

#### 2.1.1. Traditional TEC Thermal Cycling Mode

As shown in Figure 2a, the traditional TEC mode heats the PCR reaction mixture using a Peltier module [26]. The thermal resistance in this system is composed of the following components: the thermal resistance between the PCR tube and the reaction mixture $R_{s}$, the contact thermal resistance between the PCR tube and the reaction chamber $R_{t}$, the thermal resistance between the PCR chamber and the silicone grease $R_{1}$, the thermal resistance between the silicone grease and the TEC $R_{2}$, the combined thermal resistance of the TEC R, the thermal resistance between the TEC’s bottom surface and the silicone grease $R_{3}$, and the thermal resistance between the silicone grease and the heat sink $R_{4}$ [27]. These thermal resistances significantly affect the efficiency of thermal cycling.

Commercially available PCR systems typically use a Peltier module to control the temperature of a metal block. However, the thermal response of the system is significantly slower than the heating and cooling capabilities of the Peltier module itself, indicating a notable bottleneck in heat transfer. To improve efficiency, a novel design [28] that directly uses the Peltier module as a heat exchanger reduces thermal resistances $R_{1}$ and $R_{2}$ to enhance the system’s thermal response, as shown in Figure 2b. Additionally, reducing the contact thermal resistance $R_{t}$ will further improve heat transfer efficiency. For example, the traditional 0.2 mL PCR tube is redesigned as a thin plastic chamber to increase its contact area with the TEC, thereby reducing thermal contact resistance. One possible design is a serpentine-shaped, thin (0.75 mm) polycarbonate PCR microchamber to achieve this goal [29]. To further enhance heat transfer, a compact thermal cycling component with dual Peltier thermoelectric elements could be applied to the upper and lower surfaces of the microfluidic chip reaction chamber [30]. Due to the high thermal conductivity of silicon (Si), replacing traditional plastic PCR tubes with Si-based PCR chambers improves heat transfer [31]. However, native silicon inhibits PCR reactions, and coating the silicon surface with silicon dioxide (SiO_2_) is required to provide stable amplification results [32]. Building on the previous research, Zhang et al. optimized the thermal and fluidic design of Si-based chips by using a PID algorithm to control the TEC, accelerating the thermal cycling process. This chip can simultaneously test up to four samples and detect COVID-19 with 40 PCR amplification cycles in under 5 min [33]. Similarly, metals such as aluminum can be used to fabricate the reaction chamber due to their excellent thermal conductivity [34]. By employing Peltier-based thermal cycling, the overall PCR reaction time is reduced to 27 min. Given the high contamination risk of PCR consumables after amplification, addressing the recycling of consumables is crucial. One solution is to use core–shell bead micro-reactors (20 µL) for sample dispersion, encapsulating 2 µL of PCR reaction liquid to undergo thermal cycling via TEC [35]. This approach significantly reduces plastic waste while maintaining a reasonable error margin compared to traditional PCR.

Significant progress has been made in TEC-based PCR technology. However, challenges such as relatively high system costs, the need for improved temperature cycling efficiency, and power fluctuations due to environmental temperature changes persist. While the discussed TEC improvements help enhance thermal cycling efficiency, integrating the TEC with the heating chamber increases the cost and complexity of production. Furthermore, using materials like Al and Si for the heating chamber raises significant biocompatibility concerns, complicating reagent design. Although surface treatments can address some of these issues, the added complexity increases the cost of consumables per use. The optimization of the TEC model should still be based on the existing PCR tube design, as the performance, biocompatibility, and cost-effectiveness of PCR tubes have already been widely recognized and validated.

From an engineering perspective, the TEC thermal cycling mode has been widely applied in commercial PCR devices and is one of the earliest engineered technologies. Several major outbreaks of respiratory infectious diseases have driven the widespread adoption of TEC-based PCR technology in hospitals. Consequently, during peak respiratory infection seasons, hospitals typically rely on TEC-based PCR diagnostic systems. This thermal cycling technology has laid the foundation for the development of subsequent PCR technologies. Building on this platform and integrating intelligent and automated solutions, we can develop large-scale, high-throughput PCR detection systems to meet the needs of hospitals for mass screening of respiratory infectious diseases. Since TEC technology and its related consumables have been industrialized on a large scale, both the equipment and consumables offer better cost advantages, making it one of the preferred technologies for the hospital-side component in building economical, safe, and rapid diagnostic solutions for respiratory infectious diseases.

#### 2.1.2. Impedance Joule Heating Mode

Impedance Joule heating is the process by which electrical energy is converted into heat as current flows through a conductor (Figure 2b). This is one of the simplest methods for controlling temperature (above room temperature), as heat can be directly generated from electrical power and controlled by applying a voltage. As shown in the thermal resistance model in Figure 2b, both $R_{1}$ and $R_{2}$ are zero, but the resulting heat dissipation requires natural convection, which can be accelerated by using a fan.

Metal films are commonly used in impedance heating due to their excellent electrical conductivity, thermal conductivity, and durability. Materials such as platinum (Pt) [36], gold (Au) [37], aluminum (Al) [38], silver (Ag)/chlorofluorocarbon (CF) [39], nichrome (Ni)/chromium (Cr) [40], graphene [41], and light-induced graphene (LIG) [42] can be deposited as metal films on the substrate to create a thermal cycling system for heating the PCR reaction chamber. One challenge in this process lies in ensuring the adhesion between the metal film and the substrate, which can be improved by using Cr [37]. During cooling, a fan system [43] or a heat dissipation array positioned on the backside of the metal heating film [38] can be employed to accelerate heat dissipation. Additionally, surface-mount resistors (SMDs) commonly used on traditional PCBs have also been applied in the development of thermal cyclers [44].

The most successful commercialization of impedance heating is exemplified in the design by Belgrader et al. [45], which features a flat thermal cycler composed of two ceramic heating plates. These ceramic heaters, made from Al_2_N_3_, use resistive thin films patterned onto the plates. This system offers a much smaller volume compared to traditional thermal cyclers and accommodates reaction volumes up to 100 µL.

To address the contact resistance $R_{t}$ issue in the impedance heating mode, Heap et al. [46] utilized the electrolyte resistance of the PCR solution for heating and temperature monitoring. Since PCR amplification solutions are conductive, they can be heated by applying a current through them and monitored for temperature by measuring the resistance of the solution. Cooling is achieved through forced air convection at ambient temperature. The heating and cooling rates can reach 20 °C/s, with 35 PCR cycles completed in less than 12 min, producing yields comparable to traditional thermal cycling methods.

From an engineering perspective, depositing metal films on microfluidic chips increases the cost of each chip, the use of Cr to address the adhesion issue between the metal film and the substrate, while effective, undoubtedly increases the complexity and cost of the manufacturing process, making the widespread application of this technology in engineering more challenging. In practical applications, ceramic heaters have shown demonstrated potential, but, like all impedance heating methods, cooling is limited to natural heat dissipation. The smaller size of the thermal cycler offers advantages, but the cooling efficiency is lower compared to TEC-based systems. Additionally, it is possible to heat the solution by directly applying a current to the electrolyte, but the engineering challenge lies in the risk of cross-contamination due to electrode contact with different reaction solutions. Challenges such as the uniform distribution of the sample solution and varying environmental temperatures further complicate the application of this method. Despite these challenges, the impedance Joule heating mode designed by Belgrader et al. [44] has now been successfully integrated into POCT for respiratory infectious disease detection, providing technical support for community-based respiratory infection testing. This POCT technology has been widely used in respiratory disease detection in Europe, North America, and other regions, with an increasing number of manufacturers leveraging this technology to develop POCT devices, leading to its widespread adoption globally. Therefore, the impedance Joule heating mode technology is suitable for POCT and can serve as one of the preferred options for the community component in building economical, safe, and rapid diagnostic solutions for respiratory infectious diseases.

#### 2.1.3. Electromagnetic Wave Direct Heating Mode

Electromagnetic wave direct heating (EWDH) utilizes electromagnetic waves to interact with water molecules or ions in the solution, converting electromagnetic energy directly into heat. This process enables temperature cycling for thermal reactions. Unlike traditional TEC heating modes, where heat transfer is hindered by various thermal resistances such as $R_{s}$, $R_{t}$, $R_{1}$, $R_{2}$, R, $R_{3}$, and $R_{4}$, electromagnetic wave direct heating eliminates most of these resistances. The only new thermal resistance introduced is the resistance at the interface between the solution and the electromagnetic wave energy exchange, denoted as $R_{m}$. Common methods of electromagnetic wave direct heating include microwave, infrared, and laser heating (Figure 2d).

##### Microwave Heating

Microwaves are electromagnetic waves with frequencies ranging from 300 MHz to 300 GHz. Microwave heating refers to the process where microwaves interact with the PCR reaction chamber and the solution, generating heating effects. The temperature generated varies depending on the frequency and microwave power used [47]. In 2002, Fermerz et al. [48] first employed microwave heating at a frequency of 2450 MHz to heat a 0.5 mL polypropylene PCR reaction tube. Using a reaction volume of 10 µL, the reaction underwent 25 thermal cycles in 60 min. Building on this, microwave heating was applied to 2.5 mL of solution to validate the feasibility of PCR, completing 33 cycles in 2.7 h with expected amplification results [49].

Additionally, various microwave frequencies have been explored for thermal cycling. For instance, an 8 GHz frequency microwave was used for direct heating of glass microfluidic chips, coupled with cooling via cold air, which enabled 28 cycles to be completed in 42 min, a significant reduction in time compared to the previous system that took 127 min for 33 cycles [50]. In multiplexed detection, a cycle instrument powered by a 6 GHz microwave performed parallel DNA detection, achieving heating and cooling rates of approximately 7 °C/s and 6 °C/s, respectively, for a 4.1 µL reaction system [51]. Commercial microwave components commonly used in wireless communication can also generate 5.5 GHz microwave signals to heat 1 µL reaction chambers, achieving heating rates of up to 40 °C/s [52]. As the reaction volume decreases, heating of individual nanoliter-sized droplets requires precise high-frequency resonance, demanding high precision in the manufacturing of resonators. A 3 GHz microwave heating system was able to raise the temperature of droplets from room temperature to 42 °C in just 5.62 milliseconds [53].

Martinic et al. [54] developed a microwave (MW) heater based on the complementary split-ring resonator (CSRR) principle, achieving efficient microwave heating at a frequency of 2.227 GHz. With heating powers of 2.7 W and 1 W, the system heated a 4 µL sample at rates of 21 °C/s and 7.6 °C/s, respectively. By adjusting the microfluidic channels and the shape of the non-radiative CSRR, better temperature uniformity and larger volumes could be achieved, making it suitable for PCR applications. This system achieved PCR amplification in a 5.4 µL microfluidic reaction chamber using a 3.75 GHz microwave heater [55]. In a report, Pal et al. [56] introduced a standard microwave oven combined with a dry bath as the heating device for DNA extraction, replacing the water bath. This approach significantly reduced the cost and complexity associated with microwave heating.

From an engineering perspective, microwave heating offers a novel approach and technical feasibility for the development of thermal cyclers. However, the generation of microwaves requires complex hardware setups, and the design of the hardware and control systems increases both the cost and size of the instrument. Compared to existing TEC-based instruments, this does not provide clear advantages in compactness. Furthermore, localized “hot spots” created by microwave heating at high temperatures may irreversibly denature the protein structure of the polymerase, compromising its enzymatic activity. High-frequency microwave emissions present significant challenges in electromagnetic compatibility, especially in complex environments like hospitals, which complicates the practical application of this technology for PCR. The issue of heat dissipation in microwave systems also requires attention, as relying on natural cooling methods would significantly limit their engineering feasibility. Therefore, microwave heating is unlikely to become the mainstream solution for thermal cycling in the molecular detection of respiratory infectious diseases. In the short term, it is challenging to position this technology as a viable option for economical, safe, and rapid detection solutions.

##### Infrared Heating

Infrared (IR) heating relies on infrared radiation, with a typical wavelength range of 0.75 to 1000 microns. IR radiation directly penetrates the PCR reaction tubes, heating the solution without depending on the conductive process of the material. Oda et al. [57] first utilize tungsten lamps as a low-cost source of infrared radiation for the thermal cycling of PCR mixtures (5–15 µL) in a glass chamber. Cooling was achieved using a compressed air source controlled by an electromagnetic valve, with a heating rate of up to 10 °C/s and a cooling rate of up to 20 °C/s, enabling a cycle time as fast as 17 s. Building on this previous work, further research extended infrared-mediated thermal cycling to capillaries [58], with volumes as small as 160 nL. This method combined the advantages of infrared-mediated sample heating with the rapid cooling capabilities of capillaries, offering a higher surface-to-volume ratio that enabled ultra-fast PCR thermal cycling, with typical cycle times of only a few seconds.

To enhance the heating rate, a parabolic gold mirror was placed above a microfluidic chip [59], enabling infrared heating of 550 nL PCR mixtures, completing 30 cycles in just 18.8 min. Due to silicon’s low absorption of most infrared wavelengths and its excellent thermal conductivity, researchers designed an infrared-compatible Si reaction chamber [60] that could also address heat dissipation issues. Additionally, infrared-mediated thermal cycling has been integrated with low-background fluorescence detection, enabling real-time monitoring during the infrared heating process [61]. IR heating has also been applied to microfluidic systems for infrared-mediated RNA isothermal RT-PCR platforms [62], where infrared LEDs serve as the heat source, completing isothermal amplification in 70 min.

From an engineering perspective, implementing infrared heating requires the construction of a complex optical system, including light sources, filters, and optical components to control the direction of light transmission and ensure stable infrared radiation. Moreover, adapting this system to support high-throughput applications with multiple PCR reactors on a single device presents significant challenges. Since most engineered PCR technologies use real-time fluorescence-based quantification rather than electrophoresis, ensuring the compatibility of the fluorescence detection system with the optical heating system is a substantial challenge. Furthermore, infrared heating still relies on natural heat dissipation, which can impact the cycle time and efficiency of PCR. These factors present considerable engineering challenges for the widespread adoption of infrared heating in PCR systems. The infrared heating cycling mode faces several challenges and is not suitable for high-throughput bulk processing applications, so it is unlikely to be engineered for large-scale use in the short term. However, in the context of POCT, it provides a new model and approach for photothermal conversion, making it one of the potential technological options for building economical, safe, and rapid diagnostic solutions at the POCT end.

##### Laser Heating

The fundamental principle of laser heating in PCR solutions is the rapid increase in temperature within the targeted area through the high energy density of the laser. The laser beam interacts with the molecules or ions of the PCR reaction solution, converting light energy into heat, thereby heating the PCR reaction mixture. Laser heating can rapidly heat small volumes of reaction liquid without direct contact with the sample, avoiding the formation of temperature gradients and providing more precise temperature control. The wavelength of the heating laser typically falls within the infrared range, such as 1460 nm [63,64,65], 1480 nm [66], 1450 nm [67], or 1550 nm [68].

Kim et al. [63] demonstrated the use of low-power (approximately 30 mW) laser radiation as a light heating source for high-speed real-time PCR in nanoliter droplets, successfully completing 40 PCR cycles within 370 s. Furthermore, infrared laser heating has also been applied to disposable culture dishes for real-time PCR studies [64]. Subsequent studies extensively explored the development of laser-heated thermal cyclers, revealing extremely high heating and cooling efficiencies for infrared laser heating. For example, a full PCR amplification process for 1–1.6 µL of PCR reaction liquid could be completed in 36 min [65], while 50 cycles of nanoliter-scale PCR could be finished in 3.5 min [66]. Additionally, the method has been used for the lysis and amplification of breast cancer cells (nL-scale liquid) in 15 min [69] and for amplifying 1 µL PCR mixtures in 12 min [67], with 50 PCR cycles completed in 24.4 min [70].

In the field of multiplex detection, Le Roux et al. [71] employed infrared lasers for thermal cycling on a compact, valve-free plastic-integrated microfluidic chip. This system was capable of performing 18-plex PCR followed by single-base resolution, enabling rapid adaptation to other detection and diagnostic tasks, such as bacterial or sexually transmitted disease testing. The PCR detection time was optimized to 45 min. In addition, Vincent et al. [68] innovatively proposed a vision for integrating laser heating, fiber-optic PCR chambers, and fluorescence detection of PCR products, aiming to create a photonic platform for real-time PCR (qPCR) and reverse transcription real-time PCR (qRT-PCR).

From an engineering perspective, both laser heating and infrared heating, being optical heating methods, share common challenges, such as the complexity of optical systems, ensuring compatibility with fluorescence-based detection systems, and addressing the high-throughput requirements for integrating multiple PCR reactors within a single system. Additionally, the limited heating area of laser systems restricts their application to smaller reaction volumes. Larger volumes can result in localized overheating, making it difficult to scale for reaction systems requiring more than 25 µL of reagent mixture, which is often needed in industrial applications. Though Vincent C et al.’s integrated platform combines complex optical systems and microfluidic components, the engineering challenges in terms of process development, yield, and throughput remain significant. Moreover, the cost per test in such a platform is likely to be high, as the entire system must be used as consumable material for each test. Therefore, considering the current demand for large-scale, high-throughput respiratory infectious disease testing, direct laser heating for thermal cycling is not ideal. However, with the further miniaturization of PCR reaction volume and laser systems, laser heating could offer certain advantages and become feasible for engineering applications. It could be applied to thermal cycling in POCT scenarios and become one of the preferred technologies for the community component in building economical, safe, and rapid diagnostic solutions for respiratory infectious diseases.

#### 2.1.4. Electromagnetic Wave-Induced Heating (EWIH)

##### Magnetic-Induced Heating (MIH)

MIH is a method that utilizes the principle of electromagnetic induction to heat conductive materials. In PCR applications, eddy currents can be induced to generate heat in metal sheets, which then heats the PCR reaction tubes in contact with them, facilitating the thermal cycling process (Figure 2e). This approach has found some application in PCR research. For instance, an electromagnetic induction secondary coil integrated with a ring-shaped metal iron plate was used in a PCR thermal cycler, achieving heating and cooling rates of 6.5 °C/s and 4.28 °C/s, respectively [72]. Another study using a copper-welded iron plate secondary induction coil for PCR thermal cycling achieved an average heating rate of 0.8 °C/s and a cooling rate of 0.5 °C/s [73]. Additionally, a heating element based on a secondary induction coil with embedded magnetic nanoparticles in a PDMS elastomeric body was developed, enabling one thermal cycle in 1 min [74].

Xie et al. [75] reported a thermal cycler that induced eddy current heating in the PCR reaction chamber using magnetic induction. Cooling was achieved by circulating antifreeze water, with thermal resistances $R_{1}$, $R_{2}$, R, $R_{3}$, and $R_{4}$ effectively reduced to zero. The cooling process introduced only a thermal resistance $R_{w}$ between the flowing water and the PCR chamber. The experimental results demonstrated an average heating rate of 14.92 °C/s and a cooling rate of 13.39 °C/s, significantly improving the heating and cooling speeds. Furthermore, the electromagnetic heating unit has been integrated into microfluidic devices and used for wireless heating of cells via an alternating magnetic field [76].

To address the contact resistance ($R_{t}$,) between the reaction chamber and the reaction tube, Ahn et al. [77] placed a bent metal heating strip inside the reaction tube. The metal strip was heated by electromagnetic induction to heat the reaction mixture (Figure 2g), but this approach focused only on the heating process. In another study, Chen et al. [78] proposed a new wireless heating method using external electromagnetic frequency modulation. This method selectively or simultaneously activates heaters in four microfluidic chip reaction chambers, offering promising features for biomedical applications. For electromagnetic induction heating, future approaches could combine the use of metal strips for magnetic induction heating [77] with antifreeze water cooling [75] to further reduce thermal resistance, enabling faster heating and cooling cycles.

From an engineering perspective, electromagnetic induction heating is straightforward, with a simple hardware control system. It is compatible with existing mass-produced PCR reaction tubes and can support varying throughput levels within a single system. Different electromagnetic induction frequencies can be applied to control each reaction chamber individually while enabling simultaneous heating and cooling. Given the wide acceptance of consumables due to their stability, biocompatibility, and cost-effectiveness, electromagnetic wave-induced heating can enhance heating efficiency without requiring changes to existing consumables. By using liquid water for cooling, rapid heat exchange between solid and liquid states facilitates fast cooling. This approach only requires a pump to circulate cooling water and thermal dissipation can be controlled via an algorithm. Therefore, the integration of magnetic induction heating with antifreeze water cooling presents a simple and efficient engineering solution, making it highly applicable in PCR systems. The industrialization of this PCR technology will enhance the efficiency of PCR testing, providing effective technical solutions for large-scale and medium-throughput respiratory infectious disease detection in hospitals. Additionally, it can be applied to the development and engineering of community-based POCT, offering timely diagnostic tools for community settings. It could become one of the preferred technologies for the hospital and community components in building economical, safe, and rapid diagnostic solutions for respiratory infectious diseases.

##### Plasmonic Photothermal Heating

Plasmonic photothermal PCR (PPT-PCR) utilizes plasmonic materials to convert light into heat. These plasmonic materials are either dispersed within the PCR reaction mixture or in direct contact with it, effectively eliminating thermal resistance $R_{t}$ and $R_{s}$. Upon exposure to an excitation light source, plasmonic materials absorb the light energy and convert it into heat, directly heating the PCR system. When the excitation light is turned off, the plasmonic materials absorb the heat, facilitating the cooling of the PCR solution (Figure 2f). Roche et al. [79,80] proposed a plasmonic heating method for PCR, where laser irradiation of gold nanoparticles was used to generate heat. By optimizing both the PCR reaction mixture and the gold nanoparticle materials, they were able to complete 30 cycles of PCR in just 54 s. Building on this, Son et al. [25] substituted gold nanoparticles with thin Au films, which enabled ultra-fast thermal cycling, achieving 30 PCR cycles with heating and cooling rates of 12.79 ± 0.93 °C/s and 6.66 ± 0.29 °C/s, respectively, in just 5 min. Subsequent studies have optimized the materials and shapes of metal nanoparticles used in plasmonic PCR. These include gold nanoparticles [79], silver nanoparticles [81], titanium dioxide (TiO_2_) nanoparticles [82], reduced graphene oxide [83], as well as modifications based on nanoparticle structures such as nanospheres [84], nanorods [85], and nanoshells [86]. Thin-film plasmonic materials have also been explored, including carbon black thin films [87], metal films [88], and TiO_2_/gold composite films [89]. Han et al. [90] reported a low-cost, rapid, and highly selective 3D plasmonic optical cavity-enhanced PCR technique. The designed 3D plasmonic optical cavity concentrated incident light near the bottom surface of the plasmonic cavity, accumulating molecules. This setup promoted rapid thermal transfer and thermophoretic flow, minimizing fluorescence quenching effects on the bare gold surface. As a result, PCR reactions were completed in just 4 min and 24 s for 10 cycles.

From an engineering perspective, PPT-PCR offers significant advantages, notably the simultaneous implementation of both heating and cooling. Additionally, existing PCR reaction tubes can be used as consumables. However, the major challenge lies in the complexity of the optical systems required to generate the laser, as well as the need for these systems to be compatible with real-time fluorescence detection. Furthermore, uniform distribution of plasmonic materials within the PCR solution is crucial, as any inhomogeneity can affect the uniformity of heating. Achieving high throughput in a single system is also challenging, as it requires a sophisticated thermal cycling system that can accommodate multiple reactions simultaneously, which adds complexity to the engineering design and scalability. And the inclusion of plasma materials in each reaction increases the cost. Currently, the engineering implementation of this technology does not meet the technical requirements for high-throughput approaches for detecting respiratory infectious diseases. Nonetheless, as laser light sources continue to miniaturize, their application in POCT becomes increasingly attractive, particularly for rapid respiratory pathogen detection. Similarly to laser-based thermal cycling devices, plasmonic photothermal PCR could become one of the key PCR technologies for POCT applications, meeting the needs of community settings in building economical, safe, and rapid diagnostic solutions for respiratory infectious diseases.

As mentioned in the previous discussion, different temporal-domain PCR technologies have their own characteristics as summarized in Table 1, with the key parameters of typical methods, including heating and cooling rates, reaction volume, DNA fragment amplification length, and their limitations. It aims to serve as a reference for selecting the most suitable technology, enabling the design of detection systems tailored to different application scenarios and needs, such as in hospitals and communities, thereby building a safe, economical, and rapid respiratory infectious disease detection system.

### 2.2. Spatial-Domain PCR

**Figure 3:**
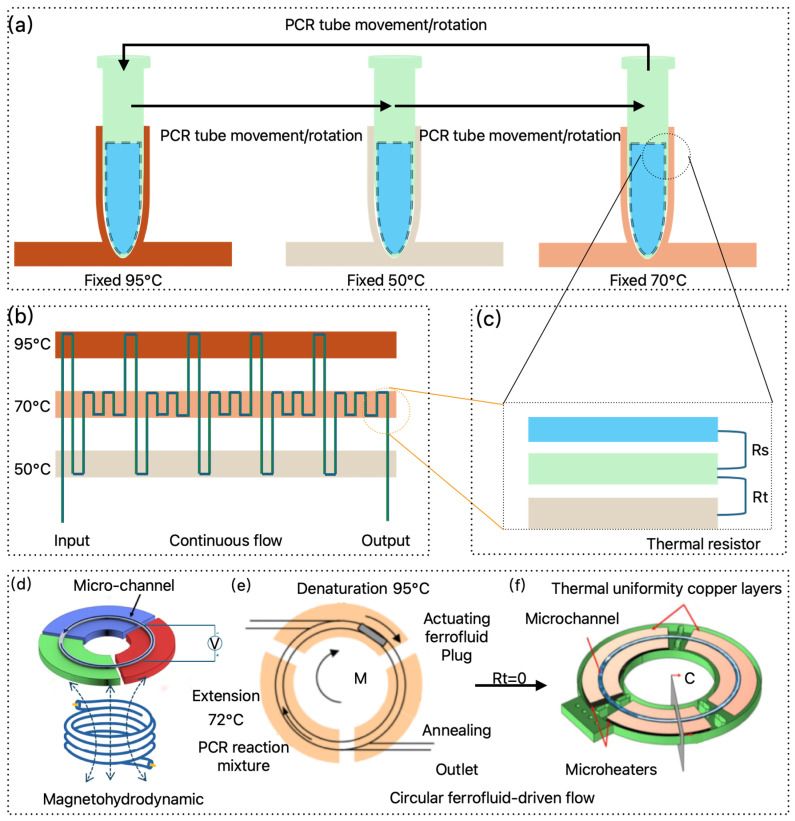
Spatial-domain PCR. (**a**) Changes in a spatial physical location; (**b**) external force-driven continuous spatial flow via a syringe pump; (**c**) equivalent thermal resistance diagram; (**d**) magnetic field-driven continuous flow; (**e**) circulating ferrofluid-driven flow (adapted from ref. [100]); (**f**) direct temperature zone contact with ferrofluid for driving flow [101].

Spatial-domain PCR involves using external forces to drive the reaction mixture through different temperature zones in space, with each zone maintaining a constant temperature (Figure 3a). This approach effectively resolves the thermal resistance issues ($R_{1}$, $R_{2}$, $R_{3}$, and $R_{4}$) found in traditional temporal-domain PCR that utilizes TEC methods. By utilizing spatial-domain PCR, only the thermal resistances $R_{s}$ and $R_{t}$ remain in the system, which enhances heat transfer efficiency from a different perspective (Figure 3c).

#### 2.2.1. Physical Space Exchange

Physical space exchange refers to the movement or rotation of the PCR reaction chamber between different temperature zones, or the movement of the temperature zones themselves, to facilitate the thermal cycling of PCR (Figure 3a). In rotational temperature control, the PCR tubes, capillaries, or microchambers are fixed, and multiple temperature blocks at different fixed temperatures rotate via a wheeled system to periodically contact the reaction chamber, enabling PCR amplification [91]. One approach for rotating PCR tubes involves using a water bath, where the reaction tubes are manually transferred between water cups set at different fixed temperatures to perform two-point or three-point thermal cycling amplification [92,93]. Automation of spatial movement has also been achieved using a rotating system equipped with two stepper motors, which provide radial and vertical motion for the rotation arm [94]. In movement temperature control, the movement of PCR tubes can also be achieved through two stepper motors, which control vertical and horizontal motions, allowing traditional PCR tubes to move through different temperature zones for thermal cycling amplification [95]. To optimize heating efficiency, increasing the contact area between the reaction tube and temperature zones is crucial. For instance, using 5 µL [95] or 3 µL capillaries instead of traditional PCR tubes reduces contact thermal resistance [96]. Additionally, PCR reaction chambers can be designed in a flat microfluidic chip form for contact with the heat source, allowing parallel movement to switch between multiple temperature zones for fast thermal cycling [97,98], this design has been successfully applied to human cytomegalovirus detection. Alternatively, the microfluidic chip can rotate to contact the temperature zones [99], facilitating the amplification of target genes. Notably, this approach has been successfully utilized to amplify the Porphyromonas gingivalis gene.

From an engineering perspective, physical space exchange requires the design of mechanical structures capable of movement or rotation. While the implementation is not overly complex, it does add to the system’s complexity and cost. A major challenge in space transformation is the lack of a thermal lid, which necessitates sealing the PCR mix with paraffin or mineral oil, adding operational complexity and hindering the miniaturization of PCR devices. Precision of movement is also crucial, the reaction tubes must consistently align with the reaction chamber, ensuring uniform insertion depth each time. Additionally, maintaining consistent contact thermal resistance during each cycle is vital. Over time, repeated motion and friction can affect the position parameters and heating units, posing significant challenges for long-term reliability and efficiency. This thermal cycling mode based on physical spatial variation enhances PCR cycling efficiency. Despite these challenges, this thermal cycling mode, based on physical spatial variation, improves PCR cycling efficiency and remains a promising solution for large-scale respiratory disease testing in hospitals. It is one of the primary technologies for hospitals to establish molecular diagnostic capabilities, helping meet the hospital’s needs for building economical, safe, and rapid diagnostic solutions for respiratory infectious diseases.

#### 2.2.2. Continuous Flow PCR

As illustrated in Figure 3b, continuous flow PCR (CF-PCR) is achieved by designing serpentine microfluidic channels within a chip, with fixed temperature points at specific locations along the loop. The sample is driven by a pump through these channels, circulating through different thermal zones, thereby enabling rapid PCR amplification. The thermal zones can be maintained using thermostated copper [102], or alternative heating materials such as indium tin oxide [103] or NiCr [104] deposited on specific regions of the substrate. In addition to serpentine channels, the flow path can also be modified into periodic spiral shapes [105] or formed by coiled Teflon tubing [106]. Beyond multiple temperature zones, a steady-state temperature gradient can be employed to create the cycling temperatures required for fluid flow [107].

Thomas et al. [108] conducted a comprehensive study identifying seven key design parameters that influence the performance of CF-PCR devices with continuous fluid flow. These parameters include the inlet and outlet positions, inlet/outlet lengths, channel spacing, aspect ratio, substrate thickness, substrate material, and temperature gradient, while also considering various practical factors. The study highlighted that the material and thickness of the substrate have the most pronounced effect on fluid temperature, offering critical guidance for optimizing thermal gradient CF cycles. To facilitate scalable, low-cost production of CF-PCR chips, researchers have employed UV roll-to-roll embossing techniques [109,110] and PDMS-glass soft lithography [111], which provide low-cost and user-friendly fabrication methods for POCT.

Despite years of research, one of the main limitations preventing CF-PCR from transitioning from the laboratory to practical applications is its lack of portability and reliance on expensive external precision pumps for sample injection. To overcome these obstacles, Li et al. [112] developed a portable CF-PCR microfluidic chip with an automated sample injection system, demonstrating promising potential for broader adoption. However, several challenges remain in CF-PCR technology, particularly related to bubble formation during thermal cycling in the serpentine micro-channels and reagent evaporation caused by the gas permeability of PDMS materials. These issues can lead to sample discontinuity and inconsistent dwell times in each temperature zone [113]. To address these challenges, Li et al. and colleagues proposed a dual-layer droplet CF-PCR microfluidic chip, which mitigates bubble formation and reagent evaporation during the thermal cycling process. Another challenge is the adsorption of proteins onto the walls of the microfluidic channels, which can be addressed by analyzing the hydrophobicity of the channel surfaces through infrared absorption spectroscopy, contact angle measurements, and surface roughness [114]. The third issue concerns the stability of the fluid flow driven by external forces. A potential solution is to stretch the Teflon tubing at the system’s outlet to four times its original length, stabilizing the flow rate when fluids of varying viscosities enter the tubing [115].

From an engineering perspective, CF-PCR requires a syringe-driven system to move the fluid back and forth, or the integration of complex microfluidic chips with mechanical forces to drive the fluid. This inherently increases both the design complexity and the development costs of the instruments and consumables. The complexity of the microfluidic chip also raises the cost of both instruments and consumables. Additionally, the issues of protein adsorption and bubble formation affect the sensitivity of the assay, and the fixed number of cycles designed for each chip limits flexibility. This inflexibility makes it difficult to adapt to different reagents and cycle requirements in engineering applications, thereby reducing the overall versatility of the system. Lastly, the compatibility with high throughput within the same system is also a challenge. Although engineering CF-PCR faces several challenges, it still holds significant potential for successful engineering. By optimizing PCR thermal cycling technology, it is possible to design low-cost cartridge-based consumables and develop POCT solutions, providing more accessible respiratory infectious disease diagnostics in community settings. It could be one of the preferred technologies for the community component in building economical, safe, and rapid diagnostic solutions for respiratory infectious diseases.

#### 2.2.3. Magnet-Driven PCR

In CF-PCR, precise syringes are required to drive the reaction mixture through different temperature zones. However, magnetically driven PCR can achieve non-contact liquid circulation in space, enabling the PCR cycling process (Figure 3d). West et al. [116] utilized a magnetohydrodynamic micropump to circulate the PCR reaction fluid through different temperature zones. This driving mechanism does not rely on pressure differences to generate flow; instead, it employs magnetohydrodynamics to effectively maintain continuous flow, which is suitable for continuous-flow reactions in circular channels. The magnetohydrodynamic driving mechanism works by applying a current to the channel, which is perpendicular to the magnetic field from below the channel, thereby generating Lorentz force to drive fluid circulation.

Sun et al. [100,117,118] proposed a ferromagnetic fluid-based driving mode, using a small ferromagnetic fluid plug that contains a liquid carrier with sub-domain magnetic particles. Under the influence of an external magnet, this plug moves along a circular micro-channel, driving the PCR mixture through three distinct temperature zones (denaturation, annealing, and extension). They also designed a high-throughput multi-channel closed-loop magnetically driven chip (Figure 3e). To minimize the contact resistance ($R_{t}$) between the reaction liquids in different temperature zones, Skaltsounis et al. [101] used printed circuit boards (PCBs) as the substrate material, integrating micro-heaters into the micro-reactors. This approach minimized the overall size of the micro-reactor and reduced the distance between the micro-heater and the micro-channel, ensuring faster heat transfer. Furthermore, using PCBs to fabricate the micro-reactors aligns with the well-established PCB industry, allowing for low-cost, reliable, reproducible, and scalable manufacturing, thereby enhancing the commercialization potential of this micro-reactor (Figure 3f).

From an engineering perspective, the above-mentioned magnet-driven approach has not been integrated with real-time fluorescence PCR functions, which may present challenges when conducting real-time fluorescence monitoring. Each reaction requires a separate injection of ferromagnetic fluid, increasing the complexity of reagent addition and operations. Additionally, an external rotating magnetic field is required to drive the movement of the ferromagnetic fluid, further complicating the instrument’s design. In practical applications, since different reagents require different reaction programs, the speed of the ferromagnetic fluid, the dimensions of the flow channel, and other parameters need to be redesigned based on the specific protocol to accommodate various experimental needs. Although magnetic-driven thermal cycling PCR technology currently faces an engineering gap compared to existing respiratory infectious disease diagnostic solutions, it offers a novel driving mechanism. In the future, this technology holds great promise for application in POCT design and the advancement of rapid respiratory disease testing. Also, it could be one of the preferred technologies for the community component in building economical, safe, and rapid diagnostic solutions for respiratory infectious diseases.

Based on the previous discussion, various techniques for spatial PCR each have their own unique characteristics. Table 2 provides a summary analysis of typical methods, focusing on parameters such as temperature ramp rate, reaction volume, amplified DNA fragment length, and associated limitations. It also provides a reference for technological approaches suitable for respiratory infectious disease detection in different application scenarios and needs, such as in hospitals and communities, thereby building a safe, economical, and rapid respiratory infectious disease detection system.

### 2.3. Spatiotemporal Unified PCR, Isothermal Amplification, and Biosensors

#### 2.3.1. Convective PCR

Following the study of temporal-domain PCR and spatial-domain PCR, researchers recognized that time and space could be unified, leading to the development of Convective PCR [119]. Convection occurs by applying a horizontal or vertical temperature gradient within a closed loop, commonly called a thermosyphon. Because the fluid elements in a convective system quickly achieve thermal equilibrium with their surroundings and pass through different temperature zones without experiencing the delays encountered in traditional methods, convective PCR enables faster cycle times [120].

**Figure 4:**
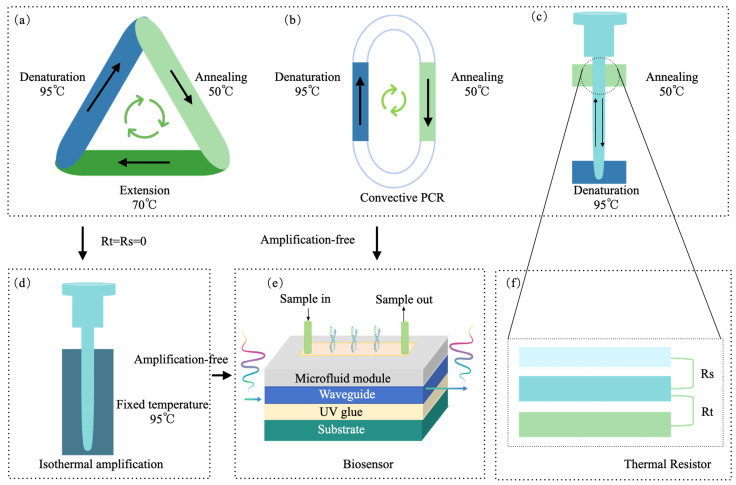
Spatiotemporal unified PCR, isothermal amplification, and biosensors. (**a**) Closed-loop convective PCR; (**b**) circular raceway convective PCR; (**c**) capillary convective PCR; (**d**) isothermal amplification; (**e**) biosensor; (**f**) convective PCR thermal resistance analysis diagram.

Convective thermal cycling loops can be designed in various shapes, including triangular loops [121,122] (Figure 4a), runway-shaped designs [123] (Figure 3b), ring-shaped loops [124] (Figure 4b), and even single-tube convective systems [125,126,127,128,129,130,131]. The single-tube system can further be classified into dual-temperature zone convection [125,126,127] or convection based on a single-point temperature gradient [128,129,130,131]. Additionally, convection technology enables the design of multi-channel convective PCR systems. Miao et al. [132] used capillary convection to design a POCT system capable of simultaneously detecting eight samples. This system was validated through repeatable detection of the A-type (H1N1) virus nucleic acid target, achieving a detection limit of 1.0 TCID50/mL, and completing the analysis in just 30 min. Khodakov et al. [133,134] developed a portable, battery-powered multi-chamber multiplex PCR system using a ring-shaped convective chamber and fluorescent quenched oligonucleotide probe microarrays. This system detected DNA targets with a sensitivity of 10 copies per sample within 30 min and was successfully applied to the detection of 20 human genome loci, 30 SNPs, and 15 bacterial strains from clinical isolates. With further optimization, Khodakov et al. showed the feasibility of simultaneous amplification and detection of 28 RNA targets from purified RNA samples within 40 min. The method used adjustable strand-displacement probes for stable detection of specific RNA species, even in high-homology backgrounds, demonstrating the capability to detect targets with up to two single-nucleotide variations. As proof of concept, this method was able to simultaneously identify seven human coronaviruses and seven SARS-CoV-2 variants in a single test.

Convective PCR offers significant advantages in terms of reduced heating and cooling cycles, facilitating much smaller thermal cycling systems. From an engineering standpoint, convective PCR holds great promise because it eliminates the need for complex thermal cycling systems and external mechanical components, making it an ideal direction for engineering development. However, several technical challenges still need to be addressed. Currently, convective PCR systems do not support real-time monitoring of the PCR reaction process or real-time tracking of cycle counts. Therefore, the design of convection loops must align with the PCR program. This indicates that PCR’s engineering direction will focus on POCT applications rather than traditional open-platform PCR systems, emphasizing the deep integration of reagents, cartridges, and instruments. Currently, most convective PCR systems utilize capillary tubes, and a major challenge is preventing air bubbles and ensuring complete sample injection into the capillary.

If convective PCR is to be further developed into a POCT system, the microfluidic chip must integrate both sample preparation (e.g., nucleic acid extraction) and the convective PCR reaction chamber into a single unit. This integration remains a critical issue in the engineering development of convective PCR. Therefore, once engineered, convective PCR will offer a highly competitive technological solution for POCT in respiratory infectious disease detection, becoming a core technological approach for meeting the future needs of communities in building economical, safe, and rapid diagnostic solutions for respiratory infectious diseases. Table 3 summarizes the key parameters of typical convective PCR methods, focusing on reaction volume, reaction time, amplified DNA fragment length, and their limitations, providing valuable guidance for selecting convective technology in future POCT applications.

#### 2.3.2. Isothermal Amplification

Isothermal amplification represents a nucleic acid amplification technique that operates at a constant temperature, eliminating the need for the cyclic heating and cooling phases required in traditional PCR to achieve different amplification stages. In isothermal amplification, only a fixed temperature needs to be maintained, removing reliance on expensive thermal cyclers. This approach unifies time and space in the amplification process. Researchers have explored various reagent detection methods for isothermal amplification, and a summary of these technologies is provided in Table 4 [135]. Rajan et al. [136] compiled various isothermal amplification techniques that can be integrated with CRISPR, offering enabling tools for future applications.

Due to its simplicity, speed, and ease of use, isothermal amplification has seen increasing application in consumer-oriented home testing. Extensive research has been conducted to explore these possibilities. Lu et al. [137] reported a rapid, low-cost, aerosol-free, and highly sensitive molecular detection method called FLASH, based on the principles of LAMP (loop-mediated isothermal amplification), using a visual lateral flow system. This method simplifies the process, making it accessible for untrained users to perform home tests. Bravo-González et al. [138] described the development of a DIY incubator for use with Eppendorf tubes, which allows for at-home detection of SARS-CoV-2. Li et al. [139] developed a portable device capable of monitoring isothermal nucleic acid amplification tests in real-time using electrochemical methods, specifically designed for home-based SARS-CoV-2 testing. Dai et al. [140] introduced a compact, pocket-sized isothermal fluorescence diagnostic IoT device suitable for home testing of SARS-CoV-2 and influenza viruses. This device weighs only 61 g and features automatic result interpretation via a smartphone, enabling seamless upload to the “EzDx cloud” for integrated health management and disease monitoring.

Cao et al. [141] proposed a CRISPR-based single-tube loop-mediated isothermal amplification method for home testing of SARS-CoV-2 RNA. Liu et al. [142] developed an integrated, instant nucleic acid screening kit that consolidates nucleic acid release, amplification, and result visualization, facilitating self-testing. The kit uses a user-friendly gaming controller interface, allowing individuals to perform tests without specialized training. Cao et al. [143] developed CoRPLA (CRISPR-regulated single-tube recombinant polymerase loop-mediated amplification), a non-amplification detection method that operates at skin temperature and is specifically designed for home use. The method has been validated with clinical samples, detecting SARS-CoV-2, RSV, influenza A virus, and HPV. The results were consistent with qPCR, demonstrating high sensitivity while eliminating false positives due to aerosol contamination. CoRPLA has successfully expanded molecular detection from laboratory environments to home use, making it suitable for routine viral infection monitoring.

From an engineering perspective, isothermal amplification is less complex than thermal cycling-based techniques, allowing for simpler instrument design. The primary challenge lies in designing isothermal amplification cartridges or chips that integrate nucleic acid release and amplification in a streamlined process. These developments aim to simplify system operation, making it more user-friendly and accessible for non-experts. On the reagent front, as isothermal amplification technologies continue to advance if their sensitivity and specificity in respiratory pathogen detection achieve parity with PCR, isothermal amplification will have vast potential for application in POCT and at-home testing. Although this provides technology to meet the need for home-based testing in building economical, safe, and rapid diagnostic solutions for respiratory infectious diseases, the risk of aerosol contamination needs to be carefully addressed.

#### 2.3.3. Biosensor

**Figure 5:**
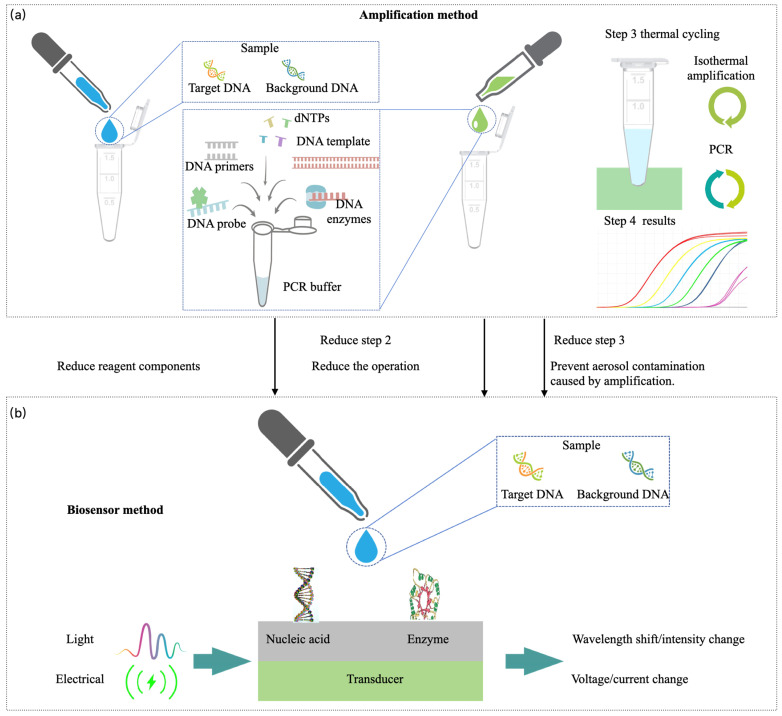
Transition from amplification-based to amplification-free technologies. (**a**) Steps and reagent components of amplification technology; (**b**) steps and sensor components of biosensor technology.

Isothermal amplification technologies represent some of the earliest attempts at home molecular detection of respiratory infections, as summarized in the previous section. However, several challenges remain in the application of amplification-based molecular diagnostics for at-home use, as shown in Figure 5. These challenges include the complexity of the reagent components required for amplification, which complicates cost reduction. Additionally, users must manage waste generated by amplification processes, which could lead to aerosol contamination. The process is not sufficiently user-friendly, and the need for numerous PCR cycles prolongs detection time. Furthermore, the accuracy of isothermal amplification methods still requires improvement.

In contrast to amplification, biosensor-based detection directly captures specific molecular targets through sensor technology and converts this information into detectable signals. Biosensor technology for antigen-based home testing saw widespread adoption during the COVID-19 pandemic, increasing public acceptance of home testing for respiratory infections. Molecular biosensors for at-home detection directly capture target molecular fragments, as shown in Figure 4e. As illustrated in Figure 5b, biosensors do not require thermal cyclers for amplification, simplifying the reagent composition and eliminating aerosol contamination caused by amplification. The absence of amplification also removes the need for positive and negative quality controls, making the process easier to operate. Moreover, as sensor technologies advance, production costs have decreased, reducing the barriers to the home use of molecular diagnostics for infectious diseases.

Molecular biosensors encompass various technologies, including optical sensing [144], electrochemical sensing [145], and micro-mechanical sensing [146]. Fozouni et al. [147] developed an amplification-free CRISPR-Cas13a detection method for directly detecting SARS-CoV-2 RNA from nasal swabs, with results readable by smartphone cameras. This method achieves a sensitivity of 100 copies/mL within 30 min and can accurately detect RNA from positive clinical samples in under 5 min. When integrated with mobile-based readers, this method demonstrates the potential for fast, low-cost, point-of-care SARS-CoV-2 testing. Song et al. developed a CRISPR/Cas13a-based catalytic fluorescence biosensor for amplification-free detection of SARS-CoV-2. This method provides quantitative detection within 50 min and has a detection limit of 18.6 copies/μL. When used for detecting SARS-CoV-2 pseudoviruses in environmental water samples, the method achieved a 100% positive detection rate, matching the results of standard RT-qPCR. Thanks to its amplification-free nature, the method has great potential for rapid SARS-CoV-2 detection in field settings and resource-limited environments. The flexibility of the CRISPR/Cas system also makes this method applicable to a variety of respiratory infectious diseases [148].

Due to its advantages, amplification-free biosensor technology is poised to play a significant role in the future of home-based detection of respiratory infections. However, challenges remain in the widespread adoption of this technology, particularly in balancing detection time, sensitivity, stability, cost, and instrument size. Future research will focus on improving detection speed without compromising sensitivity and stability, ensuring instrument size suits home use, and reducing costs. The development of artificial intelligence (AI) and advances in sensor technology and its fabrication will likely address many of these challenges.

From an engineering perspective, the main challenge lies in the fabrication of biosensors. However, as microfabrication technologies continue to evolve and mature, the production of sensors has become industrialized, leading to lower costs and easier fabrication. Given the reliance on optical and electrochemical methods, the industrialization of these technologies will support the mass production of biosensors. These combined factors will further drive the engineering and commercialization of biosensors. Once the biosensors for respiratory infectious disease detection reach commercial viability, they will become the near-perfect technology to complete home testing solutions for building economical, safe, and rapid diagnostic solutions for respiratory infectious diseases.

## 3. Full-Scenario Molecular Infectious Disease Detection Solutions

The previous sections provided an analysis and summary of the current status and development trends of various molecular diagnostic technologies. The advantages and disadvantages of each technology were explored from an engineering perspective, and the feasibility of transitioning from amplification-based to amplification-free methods was further examined.

Building on PCR technologies, isothermal amplification, biosensors, and other methodologies, and considering the unique characteristics of different application settings—such as hospitals, communities, and home environments—this section outlines tailored molecular diagnostic solutions for respiratory infectious diseases. By integrating current and future potential engineering technologies, we list here feasible candidates that are economical, safe, and rapid for infectious disease detection across diverse application scenarios.

**Figure 6:**
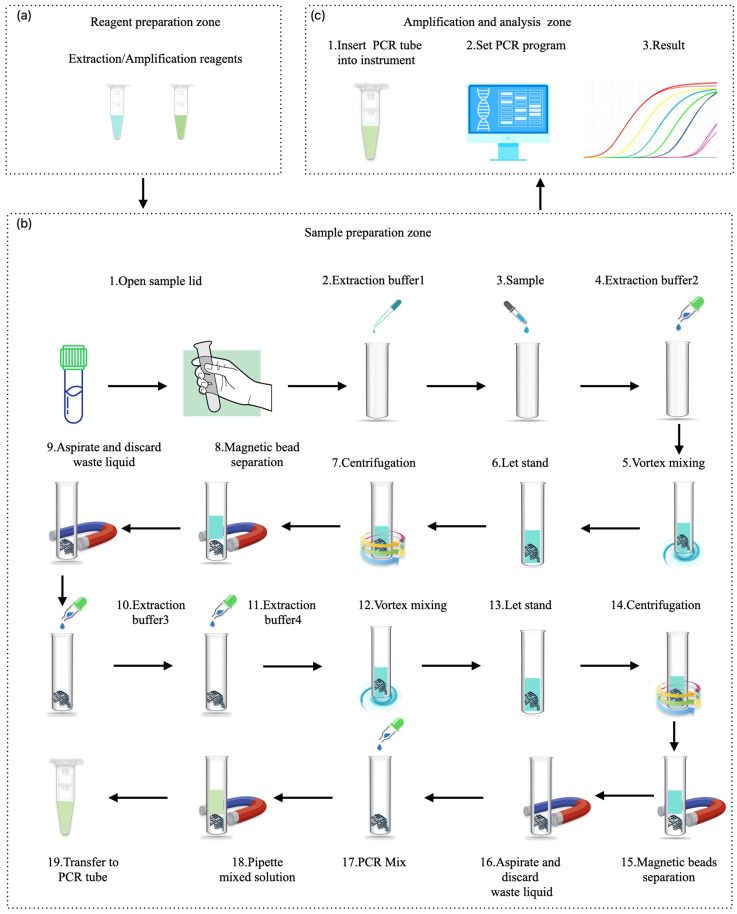
Traditional three-zone PCR laboratory in hospitals. (**a**) Reagent preparation zone; (**b**) sample processing zone; (**c**) PCR amplification and result analysis zone.

As depicted in Figure 6, traditional PCR laboratories in hospitals are typically divided into three distinct zones to minimize the risk of cross-contamination, the reagent preparation zone, sample processing zone, and PCR amplification and result analysis zone. In Figure 6a, the necessary reagents are prepared; in Figure 6b, nucleic acid extraction and PCR mixture preparation are performed; and in Figure 6c, the PCR amplification process is carried out, followed by the analysis of results and subsequent data upload for further processing.

In a hospital setting, where established PCR laboratories and stringent quality control standards are already in place, the primary focus is on ensuring the accuracy and reliability of the detection results. Given this context, the key challenge lies in optimizing current PCR technologies to achieve higher levels of automation and throughput, thereby enabling efficient handling of large sample volumes while maintaining stringent standards of accuracy and reliability. The goal is to integrate automation into existing PCR workflows, streamlining the process while preserving the integrity and precision of diagnostic results.

### 3.1. Hospital-Based Respiratory Infectious Disease Molecular Detection Solutions

**Figure 7:**
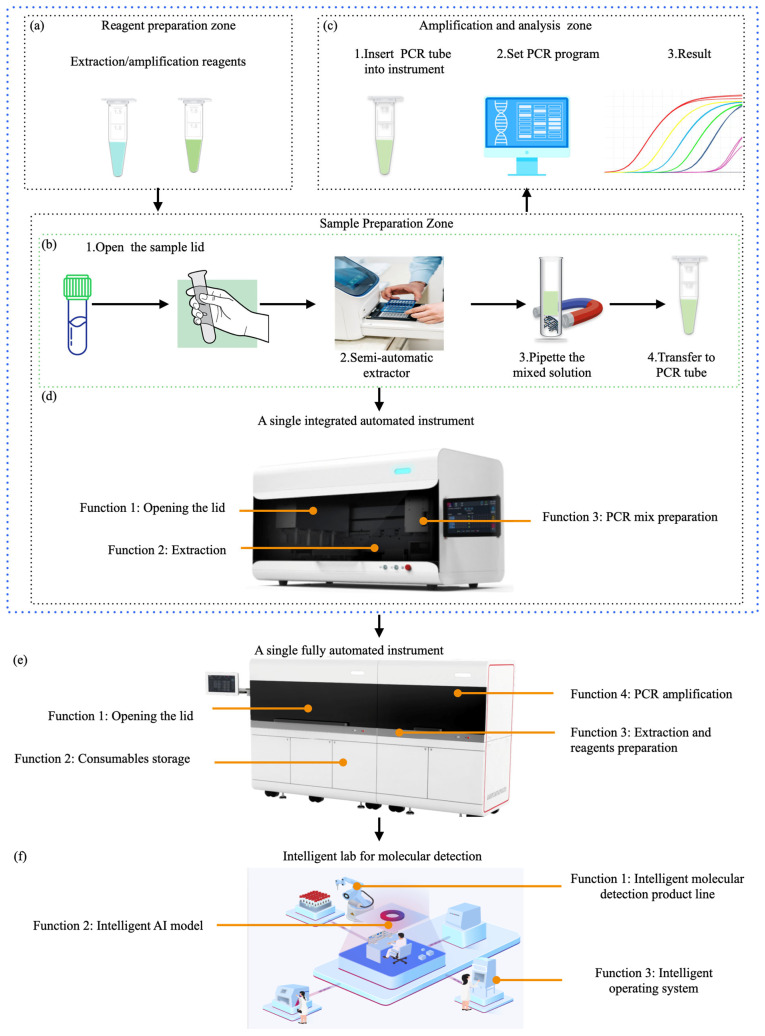
Hospital-based molecular infectious disease detection solutions. (**a**) Reagent preparation zone; (**b**) semi-automated nucleic acid extraction solution; (**c**) PCR amplification and result analysis zone; (**d**) fully automated nucleic acid extraction; (**e**) integrated molecular infectious disease detection system; (**f**) intelligent molecular infectious disease detection laboratory.

As illustrated in Figure 6, nucleic acid extraction is a complex and time-consuming process, which leads to slower detection speeds, higher costs, and affects the stability and accuracy of the results. To enable large-scale processing, several molecular detection technologies have already been engineered for automation. As shown in Figure 7b, semi-automated nucleic acid extraction systems have been developed to replace manual extraction procedure. These systems transfer the extracted nucleic acids to 96-well plates or PCR 8-tube strips for subsequent amplification and detection [149,150]. Currently, the majority of commercially available PCR systems use thermal cyclers (TECs) for amplification [151].

To further improve extraction efficiency, fully automated nucleic acid extraction systems have been introduced, as depicted in Figure 7d. These systems integrate nucleic acid extraction, sample preparation, and PCR mix construction within a single instrument, reducing the need for manual intervention [152]. However, even with full automation, the extracted nucleic acids must still be transferred to PCR plates for amplification.

To further reduce labor input and ensure the safety of personnel involved in infectious disease testing, fully integrated systems have been developed that combine reagent preparation, nucleic acid extraction, and amplification within one device, as shown in Figure 7e. Several such integrated systems are already available on the market, including Hologic Panther Fusion system [153], Roche’s Cobas series [154], and Abbott’s Alinity M [155].

In recent years, advancements in intelligent manufacturing and artificial intelligence (AI) have significantly accelerated the development of intelligent life sciences technologies. The emergence of AI-driven tools has greatly reduced manual labor and enhanced the speed of research and development [156]. Consequently, molecular diagnostics in respiratory infectious disease detection is moving towards the establishment of “intelligent laboratories” (Figure 7f). These smart laboratories integrate intelligent workflows, smart operating systems, and AI models designed for molecular diagnostics. These AI models incorporate deep perception, precise control, intelligent decision-making, quality control, and automated detection [157].

The establishment of smart laboratories will enable large-scale screening and rapid, precise diagnostics in hospital settings, meeting the high demands of large hospitals. Additionally, small-scale, all-in-one machines will allow smaller hospitals to process samples in smaller batches, helping to address the issue of tiered referrals between large and small hospitals. This integrated approach will significantly improve the efficiency, safety, and scalability of molecular infectious disease detection in both large and small healthcare settings. Thus, it will benefit the establishment of molecular diagnostic capabilities at both large and small hospital ends within the framework of economical, safe, and rapid diagnostic solutions for respiratory infectious diseases.

### 3.2. POCT-Based Respiratory Infectious Disease Molecular Detection Solutions

**Figure 8:**
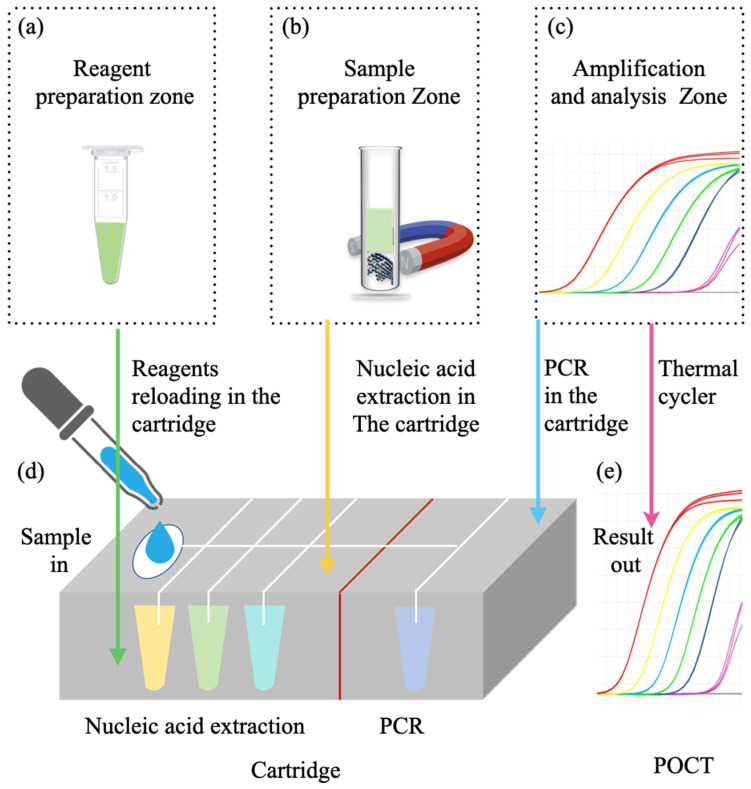
Molecular POCT system. (**a**) Reagent preparation area; (**b**) sample preparation area; (**c**) amplification and analysis area; (**d**) microfluidic cartridge; (**e**) POCT instrument.

In community healthcare settings, the absence of established PCR laboratories and trained personnel proficient in PCR techniques presents a significant barrier to the widespread adoption of molecular diagnostic technologies. Molecular POCT has emerged as a solution to this challenge. As illustrated in Figure 8, POCT leverages microfluidic technology to integrate essential steps such as reagent preparation (Figure 8a), nucleic acid extraction (Figure 8b), and amplification (Figure 8c)—typically performed in traditional laboratory settings—into a single, closed microfluidic cartridge or chip (Figure 8d). Furthermore, dedicated POCT instruments (Figure 8e) have been developed to implement a simple sample-in, result-out workflow, facilitating rapid diagnostics and reducing the barriers to molecular infectious disease testing [158].

The development of molecular POCT has seen significant progress both domestically and internationally, with many PCR technologies now being engineered for use in POCT applications. Various types of PCR technologies have been integrated into POCT systems, including the TEC heating method utilized in Sansure’s iPonatic [159], impedance-based heating PCR in GeneXpert [160], multiplex molecular POCT in FilmArray [161] using TEC heating, and continuous-flow PCR technology in QuantumNX’s QPOC [162]. In the design of POCT systems, considerations such as detection time are critical, alongside the need to accommodate low-throughput testing. This, however, results in challenges regarding increasing instrument size and the spatial constraints imposed on the testing environment.

The advent of convective PCR technology has significantly addressed this issue by reducing the size of POCT devices, thus improving portability while enhancing detection speed and efficiency. Notable commercial products incorporating convective PCR technology include Ahram Biosystems’ Palm Convective PCR [163], Taiwan GeneReach’s POCKIT Convective PCR [164], and Sansure’s iCube [165]. These systems employ capillary-driven convective PCR, as depicted in Figure 4c. The ease and portability of use inherent in POCT systems enable their deployment in community healthcare environments where PCR laboratories are unavailable, providing rapid diagnostic results that can be utilized by frontline healthcare workers. This capability is particularly crucial in emergency epidemic responses and remote areas, where POCT has demonstrated immense potential in addressing public health needs.

Moreover, with advancements in big data analytics and artificial intelligence, modern POCT systems can now be integrated with real-time network connectivity. This integration facilitates the monitoring and forecasting of respiratory infectious disease outbreaks, providing valuable data for public health authorities. The ability to link POCT devices to real-time networks not only enhances the responsiveness of diagnostic testing but also significantly improves the overall efficacy of public health surveillance, particularly in resource-limited settings. Thus, it will complete the establishment of molecular diagnostic capabilities at community ends within the framework of economical, safe, and rapid diagnostic solutions for respiratory infectious diseases. 

### 3.3. Home-Based Respiratory Infectious Disease Molecular Detection Solutions

Molecular, home-based testing refers to the use of convenient and rapid molecular diagnostic techniques (such as isothermal amplification, CRISPR, and biosensors) for patients to self-collect samples and detect infectious diseases (e.g., viral or bacterial pathogens) in the comfort of their homes. Home-based testing offers advantages such as convenience, real-time results, time savings, and reduced human contact, making it especially suitable for large-scale rapid screening of infectious diseases, such as COVID-19, influenza, and H1N1.

**Figure 9:**
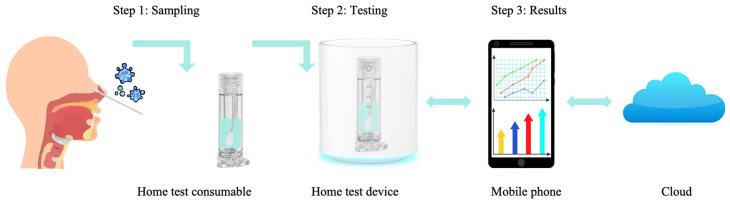
Application scenarios for home-based molecular testing products.

Figure 9 illustrates the main application scenarios for molecular detection of respiratory infectious diseases, highlighting the challenges that home-based molecular testing presents. First, since samples are self-collected by users, there is a risk of improper or insufficient sample collection, which could affect the reliability of test results. Second, the self-testing process must be simple and user-friendly, consisting of two steps—sample collection and testing—after which the results should be provided. Additionally, the proper disposal of used test materials and the potential impact of waste handling on results are concerns that require attention.

Isothermal amplification technologies gained significant traction during the COVID-19 pandemic and have already been commercialized for home testing. For example, products such as Tsure by Sansure Biotech [166] enable the detection of viral infections and allow data to be uploaded to a mobile device or cloud. Similarly, Cue Health, a U.S.-based company, offers an isothermal testing product that integrates with a mobile application, allowing users to store, review results, and even share them with healthcare providers for remote diagnosis [167].

While these home molecular diagnostic devices are simpler than POCT systems, they still involve multiple steps, especially in sample collection and processing, and require strict adherence to instructions to ensure accuracy. Moreover, solutions for managing waste and preventing aerosol contamination remain insufficient. Additionally, the detection speed (15–30 min) could be improved for a better user experience, and the accuracy and stability of results still require further refinement. For instance, the accuracy of Cue Health’s product was questioned by the FDA due to risks associated with its diagnostic accuracy [167].

Given these limitations, there is a pressing need to redesign home-based molecular diagnostic products, leveraging the non-amplification features of biosensors to address the aforementioned challenges. Biosensor-based systems are particularly promising in overcoming issues related to sample collection, waste disposal, and aerosol contamination, as they do not require amplification and thus avoid the associated contamination risks. For example, biosensor products from BiologyWorks in the U.S. have been designed for COVID-19 detection [168,169], but further updates on this product have not been reported. While biosensor-based molecular testing for respiratory infectious diseases is still in its early stages, the continued development of biosensor technology holds the potential to significantly reduce testing times and improve accuracy.

Moreover, by leveraging artificial intelligence (AI) and big data, biosensors could offer more precise results, ultimately enabling widespread use of biosensor-based home molecular testing for infectious diseases. Thus, biosensors have the potential to complete the establishment of molecular diagnostic capabilities at home within the framework of economical, safe, and rapid diagnostic solutions for respiratory infectious diseases.

Through the analysis of molecular diagnostic solutions for different scenarios, it is evident that different application environments have varying requirements in terms of throughput, detection speed, and operational convenience. Table 5 provides an overall summary of typical products, covering typical products for large hospitals, small hospitals, community settings, and home testing. This preliminary framework forms a comprehensive, economical, safe, and rapid diagnostic solution for respiratory infectious diseases across all scenarios.

## 4. Conclusions and Outlook

This review provides a comprehensive analysis of existing molecular diagnostic technologies for infectious diseases, categorizing PCR-based methods into temporal-domain and spatial-domain PCR. Temporal-domain PCR includes TEC mode, Joule heating mode, electromagnetic wave (e.g., microwave, infrared, laser) direct heating, and electromagnetic-induced heating (e.g., photothermal effect, magnetic induction heating). It summarizes typical techniques in terms of reaction systems and temperature cycling rates. Spatial-domain PCR encompasses physical space exchange modes (e.g., mobile temperature zones or mobile reaction chambers), continuous flow of liquid through different temperature zones, and electromagnetic force-driven methods (direct or mediated driving). A comparative analysis of these technologies focus on reaction systems and thermal cycling speeds. The paper further discusses convection PCR technology from a spatiotemporal perspective, examining the shapes of convection chambers (triangular, circular/track-like, single-chamber) and dual-point heating strategies for single-chamber systems, utilizing temperature gradient distribution for single-point heating. Then, isothermal amplification techniques are summarized and compared, followed by an analysis of infectious disease molecular biosensor technologies for infectious diseases, focusing on non-amplification methods.

Furthermore, this review discusses the future engineering applications of various PCR, isothermal amplification and biosensor technologies, analyzing their advantages, disadvantages, and the technical challenges encountered during the engineering process. The insights provided offer valuable guidance for the further development and practical implementation of PCR technologies, contributing to the broader adoption of molecular diagnostic techniques.

Based on the demands of various application scenarios, the review examines molecular diagnostic solutions across different levels, including large hospitals, small hospitals, communities, and home settings. It compares equipment performance across these scenarios, addressing parameters such as detection throughput, time, and technology used. This provides a reference for the future development and deployment of diagnostic solutions. By selecting appropriate molecular diagnostic technologies based on the different needs at each level and establishing diagnostic capabilities within the tiered healthcare system, an economical, safe, and rapid diagnostic solution for respiratory infectious diseases will be largely realized. As technology continues to advance and develop, this system will become more refined and operationally robust.

With the advancement of AI technology, the future development of PCR technology can be accelerated.

Firstly, by studying thermal resistance and thermal inertia, and considering the requirements of rapid heating and cooling, along with biocompatibility, AI technology can be employed to train models and make predictions to analyze and optimize material structures, thereby developing advanced functional materials, such as plasmonic particles and materials with low thermal inertia. In PCR reaction tube material research, AI can assist in identifying materials with lower thermal resistance, reduced costs, and superior biocompatibility through learning-based predictions.

Secondly, after selecting models and materials, the heating and cooling rates of the PCR thermal cycler can be optimized using AI in combination with the PID algorithm. By dynamically adjusting the heating and cooling strategies according to different thermal cycling models, AI can further fine-tune the rates, ensuring more accurate and efficient temperature changes.

Next, PCR parameters are typically fixed throughout a single run, usually using the same cycling parameters. However, as the PCR reaction progresses, the initial DNA amount is low, and the polymerase activity is high. As the cycles advance, the DNA number increases exponentially, while polymerase activity decreases significantly. To optimize the PCR reaction, the fluorescence intensity can be monitored during the process, and the denaturation, annealing, and extension step times and temperatures can be dynamically adjusted. These changes can then be fed back into the system to adjust PCR parameters, thereby achieving a more efficient reaction. AI algorithms can be employed to evaluate the final PCR result and performance, automatically adjusting the cycling conditions, thus achieving intelligent PCR.

Moreover, by real-time monitoring sample types, concentrations, and reaction processes, primer concentrations, template concentrations, and cycling times can be automatically adjusted. AI algorithms can also optimize detection curves and determine the appropriate time points, thus increasing the detection speed.

Finally, by applying AI model training to signal processing, fluorescence signal collection can be enhanced, and real-time intelligent judgment can be made for curves that are prone to human error. Based on the PCR process monitoring training model, contamination samples can be automatically detected, triggering alerts. With model training, further intelligent quality control can be implemented, avoiding the tedious process of manual quality control for each batch of liquid detection reagents, ensuring the accuracy, reliability, and efficiency of the detection process.

In summary, the application of AI technology to improve the underlying heat conduction technology of PCR and dynamically optimize the algorithms of thermal cyclers allows for automatic adjustment of annealing and extension times based on real-time PCR reaction data, as well as the optimization of mold and primer concentrations, thereby improving PCR reaction efficiency and reducing detection time. Additionally, AI can enhance fluorescence detection signals, intelligently optimize curve interpretation, automatically identify aerosol contamination, and implement intelligent quality control. These innovative methods significantly improve the speed of PCR testing while effectively reducing detection costs.

**Figure 10:**
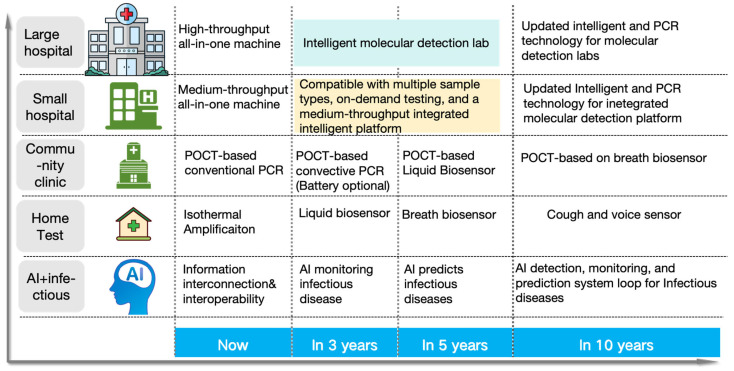
Development trends of molecular diagnostic technologies for respiratory infectious diseases in various application scenarios.

Biosensor technology plays a crucial role in molecular detection due to its rapid detection capabilities and amplification-free operation. Currently, the development of biosensors is expanding towards the detection of liquid samples, respiratory gases, and even patient coughs and sounds. For example, liquid biosensors, breath gas sensors, and cough/sound sensors are expected to be widely used in the next 3, 5, and 10 years, respectively (Figure 10). To achieve this goal, two key issues need to be addressed: first, enhancing the research on the signal conversion ability of biosensors and improving the signal-to-noise ratio, while using AI algorithms to correct signal drift and enhance signal recognition accuracy; second, utilizing liquid biosensors deployed in households to collect detection data of liquid samples from different diseases, breath gas sensors to gather gas data related to diseases, and cough/sound sensors to collect sound sample data related to diseases. By integrating real-time disease prevalence data, the family health status, and the user’s electronic medical records, big data models can be optimized and established to enable intelligent detection and prevention of respiratory infectious diseases. Given the quick, convenient, and low-cost nature of breath and cough detection, real-time monitoring of infectious diseases in households becomes much easier, making it an effective preventive tool while significantly reducing the risk of cross-infection. As a result, they become the ideal solution for home-based diagnostics in the respiratory disease detection framework proposed in this review.

With the continuous advancement of PCR and biosensor technologies, community-based POCT devices are expected to evolve into more compact and portable solutions, thereby enabling broader applications in resource-limited settings. Currently, POCT faces challenges such as high consumable costs, reliance on grid power, and large device sizes. To address these issues, within the next three years, efforts will focus on redesigning consumable structures to reduce costs, innovating thermal cycling modes to lower power consumption, and reducing the size of consumables and thermal cycling units. Simplifying mechanical components will enable the creation of battery-powered, compact, and cost-effective POCT systems. In five years, liquid-based biosensors are expected to be developed and applied to POCT, requiring further research into molecular biosensing capture mechanisms, factors affecting background signal interference, and weak signal conversion, as well as efforts to advance the industrialization of sensor manufacturing processes. By the end of the next 10 years, with advancements in microfabrication technologies, biosensors based on respiratory gas detection are expected to be widely used in POCT. Research into factors influencing weak signal conversion will be crucial. To facilitate POCT adaptation, existing large biosensors will need further downsizing and optimization. With the advancements of POCT technology, detection speed will continue to improve, and costs will decrease further. Optimizations in portability, size, and power supply options, along with the application of AI-based intelligent control models, significantly reduce the costs associated with regular quality control. These innovations will drive the broader adoption of POCT, particularly in resource-limited settings. This will significantly enhance the community-based solution within the respiratory disease detection framework.

As shown in Figure 10, high-throughput automated integrated systems in hospitals have addressed the high-throughput automation issue in batch testing, enhancing detection efficiency. However, these systems still rely on manual operation, carry the risk of aerosol contamination, and require significant quality control. With the ongoing development of AI technology, the future will gradually move towards unmanned operations, driving the intelligent transformation of laboratories. This transformation can be divided into three steps: first, integrating all laboratory equipment into the intelligent IoT; second, digitally controlling and monitoring all workflows; and third, establishing intelligent models to analyze detection results using datasets. With the open-source availability of large AI models, the intelligent laboratory’s model can be deployed locally. By training on molecular detection PCR processes, test instructions, quality control data, work environment, and test result validation, a large model for molecular detection laboratories is established. This model enables automatic recognition of sample barcodes and test reagent kits and intelligently manages consumables in the online warehouse. To prevent contamination, the system supports automatic disinfection of medical waste. Through this model, intelligent quality control is applied to test samples and data, enabling intelligent interpretation and report generation. Additionally, intelligent monitoring and analysis of the entire process will enable unmanned operation. Advancements in these technologies will further enhance the high-throughput and rapid testing capabilities at hospitals, streamline procedures for healthcare workers, reduce infection risks, and improve safety, and help lower testing costs. This will provide a high-quality solution for the hospital-based testing component of the proposed respiratory disease detection framework.

Leveraging AI and IoT technologies in the next 3 to 10 years will enable data interconnectivity between large hospitals, small hospitals, community healthcare, and home-based testing, enabling intelligent infectious disease detection, dynamic monitoring, and predictions of future disease trends. By connecting devices through the Internet of Things (IoT) and information technologies, real-time collection of raw data during the detection process can be achieved. Specialized machine learning models will be developed to identify and interpret PCR curves, allowing them to learn from experimental data and predict pathogen presence. These models will be validated and optimized based on experimental data to ensure their accuracy and reliability in real-world applications. In addition, the models will be able to automatically identify error points in the PCR curves and optimize them, making the curves closer to real data. Key technological breakthroughs in each study will include the development of new algorithms and models that can efficiently process and analyze large volumes of PCR data, improving diagnostic precision. At the same time, by classifying and analyzing various data (such as regional information, testing structures, and factors related to different levels of health institutions), precise monitoring of respiratory infectious disease trends can be achieved. This multidimensional data monitoring system helps to promptly identify epidemic trend changes across different regions and health institutions, providing real-time and scientifically grounded information for public health decision-making.

Furthermore, by collecting diverse data sources, including social media sentiment data, data from disease control centers, hospital testing data, cloud-based detection data, and population mobility data, a series of preprocessing steps such as data cleaning, deduplication, and classification will be conducted. These processed datasets will then be further analyzed using natural language processing and image data processing technologies to extract key information. By identifying crucial data points such as disease categories and geographical locations, an integrated smart epidemic prevention platform will be established. This platform will transmit and analyze the data in real time, applying advanced data analysis methods to deeply analyze the epidemic and detect patterns. It will set appropriate alert thresholds and provide gradient-based risk assessments of infectious diseases. This will offer scientific support for infectious disease prevention, epidemic trend forecasting, and emergency drug stockpiling, improving overall prevention efficiency and emergency response capabilities. It will also improve models capable of processing and analyzing large volumes of PCR data, ultimately improving diagnostic accuracy. Finally, with the advancements in IoT and AI, the respiratory disease detection solution proposed in this review integrates intelligent capabilities across the entire process, from prediction and prevention to detection. This has further strengthened the framework’s advantages in speed, safety, and cost-effectiveness.

In brief, we present here a forward-looking vision for infectious disease surveillance through an integrated, intelligent system combining advanced molecular diagnostics, artificial intelligence (AI), and Internet of Things (IoT) technologies. This comprehensive approach promises to continuously improve public health management and enhance global preparedness for future outbreaks.

## Figures and Tables

**Table 1:** Overview of the typical temporal-domain PCR technologies.

Time-Domain PCR	Disadvantages	Reaction Volume	PCR Chamber	Average Heating/Cooling Rate	Fragment Length	Ref.
TEC	The heating efficiency of TEC is low, requiring an effective cooling system, and its power output varies with changes in ambient temperature.	1–50 μL	Plastic tube	3.3 °C/s	N/A	[27]
		30 μL	Plastic tube	3 °C/s	1000 bp	[28]
		25 μL /50 μL	PC chip	30 cycles/30 min	221 bp	[29]
		10–20 μL	Glass chip	1 cycle/1.25 min	199 bp	[30]
		5.5 μL	Silicon chip	40 cycles/5 min	92 bp	[33]
Joule heater	The heat dissipation rate is slow, requiring a redesigned cooling system.	8.5 μL	Plastic chip	30 cycles/70 min	180 bp	[36]
		8 μL	Glass chip	1 cycle/1.25 min	260 bp	[37]
		10 μL	Glass chip	35 cycles/10 min	64/121/172 bp	[39]
		3 μL	NiCr/glass chip	11.6–33.3/4.1 °C/s	N/A	[40]
EWDH-microwave	Complex hardware control is required to implement microwaves.	10 μL	PP tube	20 cycles/60 min	220 bp	[47]
		0.7 μL	Glass chip	28 cycles/42 min	112 bp	[49]
		4.1 μL	PC	7/6 °C/s	N/A	[51]
		4 μL	N/A	21/7.6 °C/s	N/A	[54]
EWDH-infrared	A complex optical system is required; optical components are typically bulky; and precise mechanical design is necessary for energy focusing.	5–15 μL	Glass chamber	10/20 °C/s	N/A	[57]
		160 nL	Capillary	25 cycles/15 min	500 bp	[58]
		550 nL	Glass microchip	30 cycles/18 min	211 bp	[59]
EWDH-laser	A complex optical system is required; optical components are typically bulky; the cost of laser is relatively high; and it is difficult to heat large volumes.	20–100 nL	Hybrislip	40 cycles/6.17 min	187 bp	[63]
		1–1.6 μL	Polymeric	25 cycles/30 min	N/A	[65]
		1 μL	Polymeric chip	25 cycles/12 min	500 bp	[67]
EWIH-MIH	It requires a high-frequency power supply, which can generate noise and EMI, and demands complex hardware control and precise system design.	1 μL	Plastic tube	6.5/4.28 °C/s	N/A	[72]
		25 μL /50 μL	Plastic tube	14.92/13.39 °C/s	N/A	[75]
		250 μL	Plastic tube	6.5 J/s	N/A	[77]
EWIH-PPT	A complex optical system is required; optical components are typically bulky; and precise mechanical design is necessary for energy focusing.	10–25 μL	Plastic tube	30 cycles/54 s	250–300 bp	[80]
		10 μL	PMMA well	12.79/6.66 °C/s	N/A	[25]
		25 μL	PDMS cavity	30 cycles/12 min	113 bp	[90]

**Table 2:** An overview of the typical spatial PCR technologies.

Spatial-Domain-PCR	Disadvantages	Reaction Volume	PCR Chamber	Average Heating/Cooling Rate	Fragment Length	Ref.
Space Exchange	It requires a complex motion mechanism, and without a thermal lid, mineral oil needs to be added. It is difficult to miniaturize.	25 μL	Plastic tube	26 cycles/21 min	362 bp	[91]
		20/25 μL	Glass capillaries	15/13 °C/s	100–1500 bp	[92]
		50 μL	Plastic tube	40 cycles/13.8 min	70 bp	[94]
		5 μL	Capillary tube	1 cycle/15–60 s	70 bp	[96]
		25 μL	PC chip	13.33 °C/s	120 bp	[97]
CF	This system faces several challenges, including fixed cycle numbers, reagent adsorption on the microfluidic chip, the presence of bubbles in the micro-channels, difficulties in real-time detection, and complexities in the design and production of consumables.	10 μL	Glass chip	20 cylces/1.5–18.7 min	176 bp	[102]
		25 μL	Glass chip	1 cycle/45–90 s	450 bp	[103]
		20 μL	TOP chip	40 cycles/10–40 min	69/85 bp	[109]
		25 μL	TOP chip	40 cycles/20–50 min	120 bp	[110]
		20 μL	PDMS/glass chip	30 cycles/20.41 min	594 bp	[111]
		25 μL	Silicon chip	40 cycles/11 min	197 bp	[113]
Magnetic Drive	Magnetohydrodynamic (MHD) applications still need improvement, and the magnetic control equipment is complex.	6 μL	Silicon/SU8	1 cycle/1.5 min	142 bp	[116]
		2 μL	PMMA channel	25 cycles/4 min	500 bp	[100]
		10 μL	PCB channel	30 cycles/3 min	126 bp	[117]

**Table 3:** An overview of the typical convective PCR technologies.

Convective-PCR	Disadvantages	Reaction Volume	PCR Chamber	Equivalent Time	Fragment Length	Ref.
Triangle	The reaction chamber for PCR should be carefully designed, and the cycle count should not be recorded. There is a risk of non-specific amplification.	25 μL	FEP tubing	30 min (1 cycle/42 s)	191 bp	[121]
		21 μL	PTFE capillary	30 min (N/A cycle/s)	111 bp	[122]
Circle/track		50 μL	PP chip	20 min (1 cycle/24 s)	160 bp	[123]
		4.1 μL	COP chip	15 min (1 cycle/5.7 s)	159 bp	[124]
		25 μL	Glass/polymer	20 min (N/A cycle/s)	100 bp	[133]
Capillary (two-temperature zone)		40 μL	Capillary tubes	25 min (N/A cycle/s)	105 bp	[125]
		40 μL	Capillary tubes	25 min (N/A cycle/s)	159 bp	[127]
Capillary (one-temperature zone)		50 μL	Capillary tubes	30 min (N/A cycle/s)	67 bp	[128]
		75 μL	Glass capillary	28 min (1 cycle/30 s)	122 bp	[129]

**Table 4:** Examples of isothermal amplification techniques and their characteristics.

Isothermal Amplification Techniques	Sensitivity (Copies/mL)	Target	Reaction Time	Primers/Probes	Temperature
LAMP	10^7^–10^9^	DNA, (RNA)	1–2 h	4–6	60–65 °C
NASBA	10^6^–10^9^	RNA(DNA)	90 min	2	41 °C
SDA	10^7^–10^9^	DNA	1–1.5 h	2–4	37–70 °C
RCA	10^3^–10^7^	DNA(RNA)	1 h	2	65 °C
CPA	10^4^–10^7^	DNA	1–2 h	5	60–65 °C
EXPAR	10^6^–10^9^	Short DNA	1–2 h	N/A	60 °C
WGA	10^3^–10^7^	DNA	1 h	N/A	37 °C
RPA	10^3^–10^6^	DNA	1 h	2	37–42 °C
HDA	10^7^–10^9^	DNA	1–2 h	2	37 °C
SMART	10^4^–10⁵	RNA	2 h	2	41 °C
SPIA	10^7^–10^9^	DNA, RNA	30–90 min	1	47 °C
DAMP	10^7^–10^9^	DNA	1–2 h	6	60 °C

**Table 5:** Summary of technical solutions for various scenarios.

Scene	Product Name	Throughput	Detection Time	Detection Technology	Refs.
Large hospital	Roche cobas^®^ 8800 system (Roche Diagnostics, Basel, Switzerland)	960/8 h	N/A	PCR	[154]
	Abbot Anility M (Abbott Molecular, Des Plaines, USA)	300/8 h	N/A	PCR	[155]
	Hologic Panther Fusion^®^ system (Hologic, Inc., Marlborough, USA)	500/8 h	N/A	PCR	[153]
Small hospital	Roche cobas^®^ 5800 system Roche Diagnostics, Basel, Switzerland)	144/8 h	N/A	PCR	[154]
	Molarray MW-1600L (Suzhou Molarray, Suzhou, China)	1–16	70 min	PCR	[170]
	Autobio AutoMolec 1600 (Autobio Diagnostics, Zhengzhou, China)	192/8 h	100 min	PCR	[171]
Community clinic	Sansure iPonatic (Sansure, Changsha, China)	1–8	45 min	PCR/POCT	[159]
	Cepheid GeneXpert (Cepheid, Sunnyvale, CA, USA)	1–4	60 min	PCR/POCT	[160]
	Biofire Filmarray (BioFire Diagnostics, Salt Lake City, USA)	1–8	60 min	PCR/POCT	[161]
Homecare	Sansure Tsure (Sansure, Changsha, China)	1	15–30 min	LAMP	[166]
	Cuehealth (Cue Health Inc., San Diego, USA)	1	15–30 min	LAMP	[167]
	BiologyWorks (BiologyWorks, Inc., Los Angeles, USA)	1	<10 min	Biosensor	[168,169]

## Data Availability

No new data were created or analyzed in this study.
